# Coronaviruses Nsp5 Antagonizes Porcine Gasdermin D-Mediated Pyroptosis by Cleaving Pore-Forming p30 Fragment

**DOI:** 10.1128/mbio.02739-21

**Published:** 2022-01-11

**Authors:** Fushan Shi, Qian Lv, Tingjun Wang, Jidong Xu, Wei Xu, Yuhua Shi, Xinyu Fu, Tianming Yang, Yang Yang, Lenan Zhuang, Weihuan Fang, Jinyan Gu, Xiaoliang Li

**Affiliations:** a Department of Veterinary Medicine, College of Animal Sciences, Zhejiang University, Hangzhou, Zhejiang, China; b MOA Key Laboratory of Animal Virology, Center for Veterinary Sciences, Zhejiang University, Hangzhou, Zhejiang, China; c Institute of Preventive Veterinary Medicine, Zhejiang Provincial Key Laboratory of Preventive Veterinary Medicine, Zhejiang University, Hangzhou, Zhejiang, China; d Veterinary Teaching Hospital, Center for Veterinary Sciences, Zhejiang University, Hangzhou, Zhejiang, China; e Key Laboratory of Applied Technology on Green-Eco-Healthy Animal Husbandry of Zhejiang Province, Zhejiang Provincial Engineering Laboratory for Animal Health Inspection & Internet Technology, College of Animal Science and Technology & College of Veterinary Medicine of Zhejiang A&F University, Hangzhou, Zhejiang, China; f Hainan Institute of Zhejiang University, Sanya, Hainan, China; University of Maryland School of Medicine; Duke University School of Medicine

**Keywords:** GSDMD, Nsp5, coronavirus, pyroptosis

## Abstract

Coronaviruses (CoVs) are a family of RNA viruses that typically cause respiratory, enteric, and hepatic diseases in animals and humans. Here, we use porcine epidemic diarrhea virus (PEDV) as a model of CoVs to illustrate the reciprocal regulation between CoV infection and pyroptosis. For the first time, we elucidate the molecular mechanism of porcine gasdermin D (pGSDMD)-mediated pyroptosis and demonstrate that amino acids R238, T239, and F240 within pGSDMD-p30 are critical for pyroptosis. Furthermore, 3C-like protease Nsp5 from SARS-CoV-2, MERS-CoV, PDCoV, and PEDV can cleave pGSDMD at the Q193-G194 junction to produce two fragments unable to trigger pyroptosis. The two cleaved fragments could not inhibit PEDV replication. In addition, Nsp5 from SARS-CoV-2 and MERS-CoV also cleave human GSDMD (hGSDMD). Therefore, we provide clear evidence that PEDV may utilize the Nsp5-GSDMD pathway to inhibit pyroptosis and, thus, facilitate viral replication during the initial period, suggesting an important strategy for the coronaviruses to sustain their infection.

## INTRODUCTION

Coronaviruses are enveloped positive single-strand RNA viruses that belong to the family *Coronaviridae* (1). According to serological and genotypic characterizations, CoVs are divided into four genera, including *Alphacoronavirus* (α-CoV), *Betacoronavirus* (β-CoV), *Gammacoronavirus* (γ-CoV), and *Deltacoronavirus* (δ-CoV) (2, 3). As a member of the *Alphacoronavirus* genus, porcine epidemic diarrhea virus (PEDV) was first identified in Europe in 1971 and characterized by severe diarrhea, dehydration, vomiting, and high mortality in suckling piglets (4). The viral genome of PEDV is approximately 28 kb and encodes an accessory protein, two polyproteins, and 4 structural proteins. Most of the synthesized polyproteins are cleaved by nonstructural protein 5 (Nsp5), a 3C-like protease encoded by ORF1a, and the protease activity of Nsp5 is essential for PEDV replication (5). Nsp5 proteins from different CoVs share highly conserved amino acid sequences, which makes Nsp5 an ideal broad-spectrum antiviral target (6, 7). It has been reported that 3C-like proteases of different viruses, including foot-and-mouth disease virus (FMDV), hepatitis A virus (HAV), and enterovirus 71 (EV71), can antagonize innate immune signaling by disrupting one or more components of the interferon-inducing pathways (8–12). For coronaviruses, PEDV Nsp5 antagonizes type I interferon signaling by cleaving the nuclear transcription factor kappa B essential modulator (NEMO) at Q231 (5). Porcine deltacoronavirus (PDCoV) Nsp5 cleaves the porcine mRNA-decapping enzyme 1a (pDCP1A) at Q343 to facilitate its replication (13). A recently published study demonstrates that SARS-CoV-2 Nsp5 can cleave TAB1 and NLRP12 at two distinct cleavage sites (14). Although many studies have demonstrated the immune evasion strategies of coronaviruses, the molecular mechanism between coronaviruses replication and the innate immune response needs to be further investigated.

Pyroptosis is a form of programmed cell death characterized by cell swelling, pore formation in the plasma, lysis, and releases of cytoplasmic contents (15, 16). This type of inflammatory cell death functions as an innate immune effector to antagonize pathogenic microorganisms. Recent studies have identified gasdermin D (GSDMD) as an executioner of pyroptosis upon cleavage and activation by caspase-1 and caspase-4/5/11 (15–17). The cleaved N terminus of GSDMD (GSDMD-p30) can bind to lipids and phosphatidylethanolamine to form pores 10 to 20 nm in size, leading to pyroptosis (18–20). During infection, pyroptosis helps the host eliminate infected cells and thereby restricts proliferation of viruses and intracellular bacteria (21–24). The 3C-like protease of EV71 virus is known to facilitate its replication by inhibiting pyroptosis through cleaving the active GSDMD-p30 (25). However, the relationship between coronavirus infection and GSDMD-mediated pyroptosis has not been fully illustrated.

In this study, we used PEDV as a model of CoVs to investigate the relationship between CoV infection and pyroptosis. We found that the pGSDMD-mediated pyroptosis inhibited PEDV replication. However, during the early stage of infection, Nsp5 of PEDV directly cleaved pGSDMD at the Q193-G194 junction and produced two inactive fragments. The cleaved fragments had no inhibitory effect on PEDV replication. We found that Nsp5 from other coronaviruses, such as PDCoV, SARS-CoV-2, and MERS-CoV, also had the protease activity to cleave both hGSDMD and pGSDMD. Therefore, these results demonstrated a previously unknown mechanism of coronaviruses to escape from pyroptosis.

## RESULTS

### PEDV infection induces the reduction of pGSDMD.

Since GSDMD has been reported as a key effector for pyroptosis, many studies had been performed on human and murine GSDMD, but studies focusing on pGSDMD and its function against pathogenic infection were rare. Thus, the amino acid sequence of pGSDMD was predicted and aligned with other GSDMD homologs from human and mouse (see Fig. S1 in the supplemental material), and polyclonal antibody against pGSDMD was prepared as previously described (Fig. S2) (26, 27).

10.1128/mBio.02739-21.1 FIG S1 Alignment of the amino acid sequence of pGSDMD and GSDMD homologs from human (GenBank accession no. NP_001159709.1) and mouse (GenBank accession no. 6N9N_A). Download FIG S1, JPG file, 1.4 MB. Copyright © 2022 Shi et al. 2022 Shi et al. https://creativecommons.org/licenses/by/4.0/ This content is distributed under the terms of the Creative Commons Attribution 4.0 International license.

10.1128/mBio.02739-21.2 FIG S2 HEK293T cells were mock transfected or transfected with plasmids encoding p3×Flag-N-pGSDMD-FL. At 24 h after transfection, cell lysates were analyzed by immunoblotting with antibodies for Flag, β-actin, and the polyclonal antibody directed against pGSDMD, prepared in our laboratory. Download FIG S2, JPG file, 0.1 MB. Copyright © 2022 Shi et al. 2022 Shi et al. https://creativecommons.org/licenses/by/4.0/ This content is distributed under the terms of the Creative Commons Attribution 4.0 International license.

**FIG 1:**
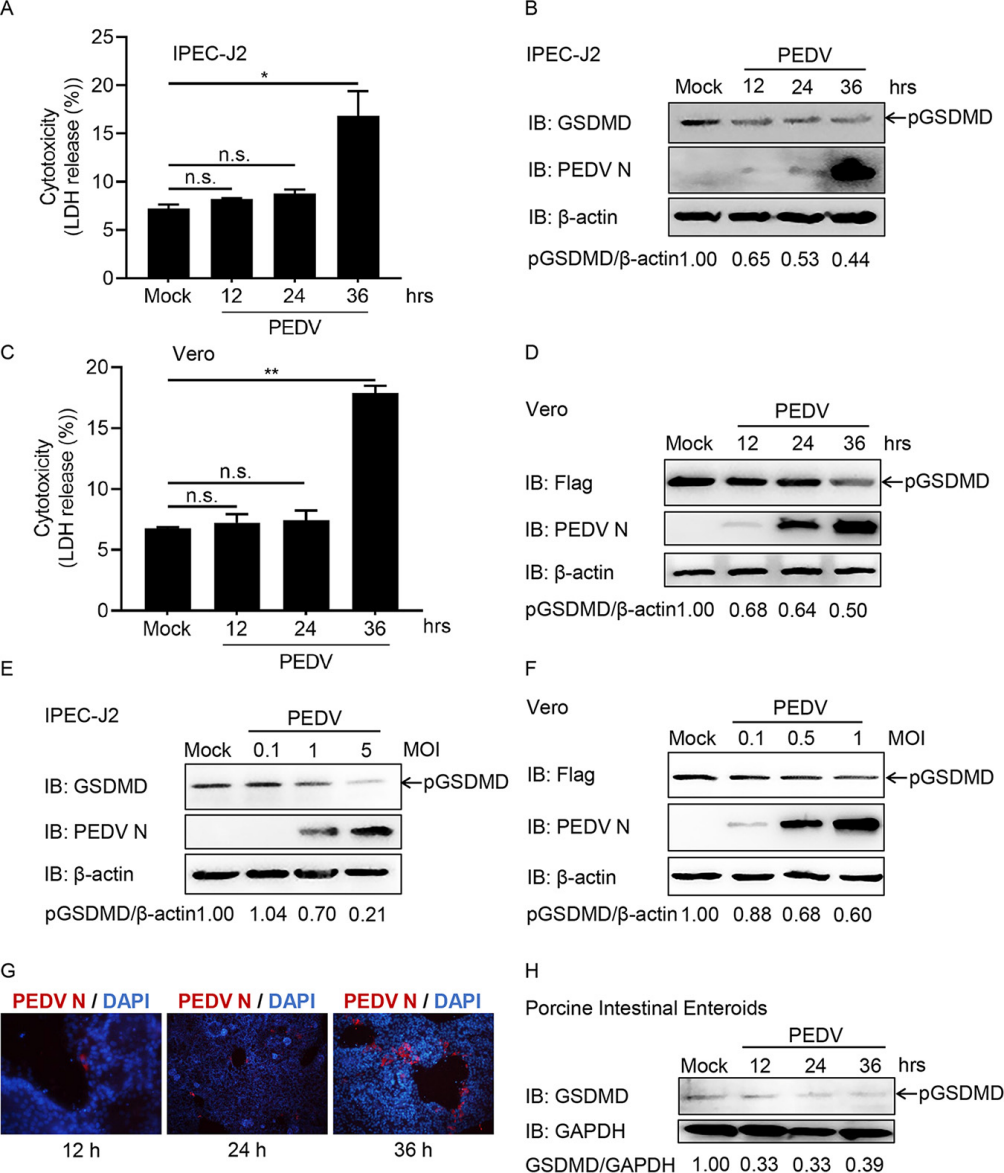
PEDV infection induces the degradation of pGSDMD. (A and B) IPEC-J2 cells were mock infected or infected with PEDV at an MOI of 1. At the indicated time points, the supernatants were collected and analyzed for LDH level (A), and cell lysates were processed for immunoblotting (IB) (B). (C and D) Vero cells were transfected with plasmid encoding p3×Flag-N-pGSDMD-FL. At 24 h after transfection, the cells were mock infected or infected with PEDV at an MOI of 0.5. At the indicated time points after infection, the supernatants were collected and analyzed for LDH level (C), and cell lysates were processed for immunoblotting (D). (E) IPEC-J2 cells were mock infected or infected with different doses of PEDV. At 24 h after infection, the cells were processed for immunoblotting. (F) Vero cells were transfected with plasmid encoding p3×Flag-N-pGSDMD-FL. At 24 h after transfection, the cells were mock infected or infected with different doses of PEDV for another 24 h, and then the cells were processed for immunoblotting. (G and H) Porcine intestinal enteroids were mock infected or infected with PEDV at an MOI of 1. At the indicated time points, the cells were immunostained (G) and lysed for immunoblotting (H). PEDV is shown in red using the antibody against viral structure PEDV N. DAPI-stained nuclei are shown in blue. The analyses were performed by one-way ANOVA with Tukey’s multiple-comparison test (not significant [n.s.], *P > *0.05; *, *P < *0.05; **, *P < *0.01).

To determine whether PEDV infection targets pGSDMD, IPEC-J2 cells were infected with PEDV at the indicated time points. Cell death was evaluated by LDH release. The results showed that PEDV infection did not cause LDH release at early time points (Fig. 1A). However, significant LDH release, representing different types of cell death, including pyroptosis, apoptosis, or necrosis, was induced at 36 h postinfection (Fig. 1A). Furthermore, PEDV infection decreased the amount of pGSDMD in IPEC-J2 cells (Fig. 1B). Similar results were observed in Vero cells (Fig. 1C and D). In addition, the reduction of pGSDMD induced by PEDV infection was multiplicity of infection (MOI) dependent in IPEC-J2 and Vero cells (Fig. 1E and F). To further confirm this, we established porcine intestinal enteroids as a PEDV infection model *in vitro*, which exhibited advantages in investigating the interactions between intestines and PEDV (28). PEDV infection did induce pGSDMD reduction in porcine intestinal enteroids (Fig. 1G and H). These results indicate that PEDV infection decreases the amount of pGSDMD.

### pCaspase-1 cleaves pGSDMD at residue D279-G280 and induces pyroptosis.

**FIG 2:**
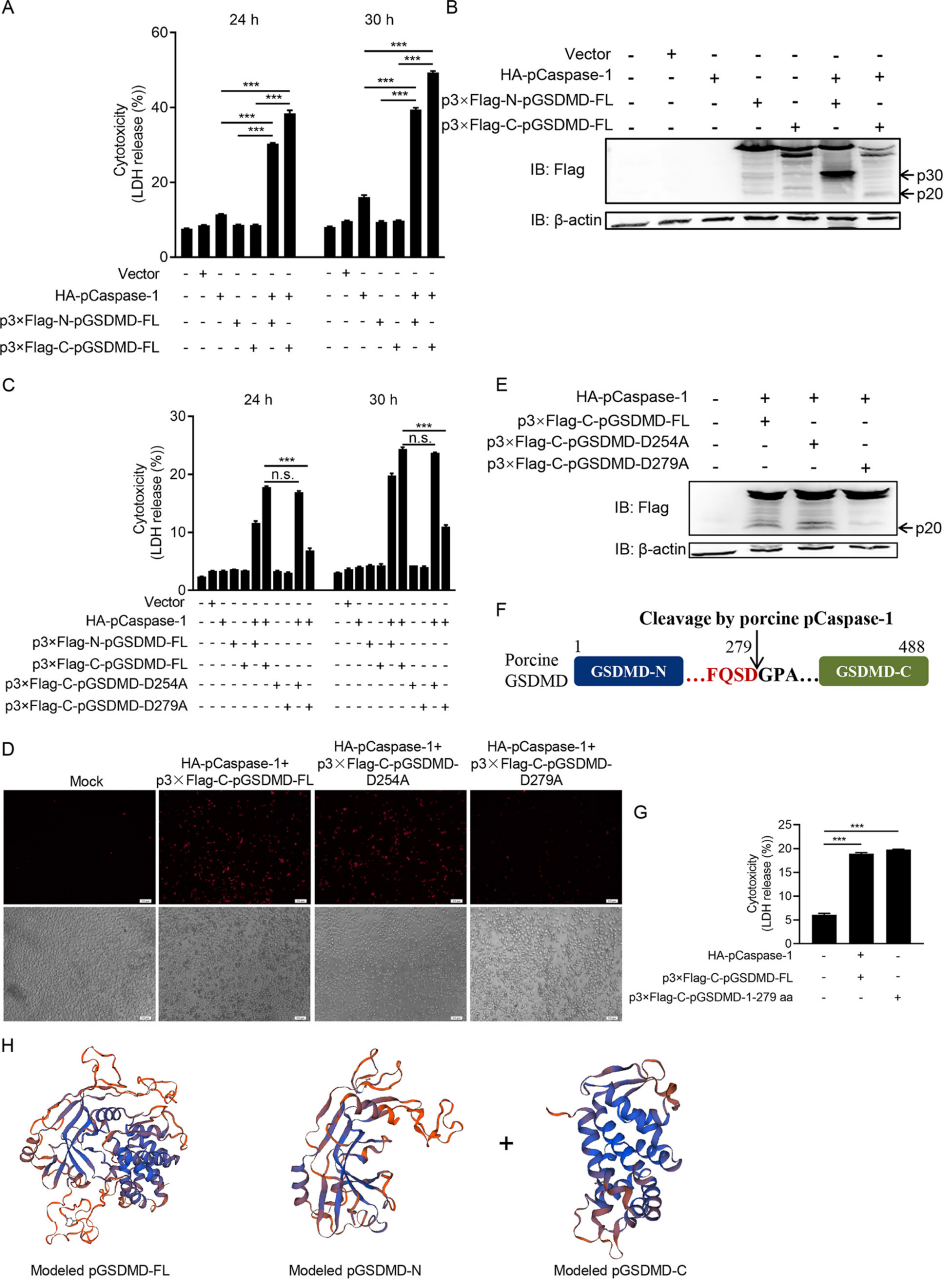
pCaspase-1 cleaves pGSDMD at residue D279-G280 and induces pyroptosis. (A and B) HEK293T cells were cotransfected with plasmids encoding HA-pCaspase-1 and p3×Flag-N-pGSDMD-FL or p3×Flag-C-pGSDMD-FL. (A) At the indicated time points after transfection, the supernatants were collected and analyzed for LDH levels. (B) At 24 h after transfection, the cells were processed for immunoblotting. (C, D, and E) HEK293T cells were transfected with plasmids as shown. (C) At the indicated time points after transfection, the supernatants were collected and analyzed for LDH levels. At 24 h after transfection, the cells were stained with PI and analyzed with fluorescence microscopy (D) or processed for immunoblotting (E). (F) Cartoon diagram of pGSDMD structure and the cleavage site by pCaspase-1. (G) HEK293T cells were transfected with plasmids encoding HA-pCaspase-1 and p3×Flag-C-pGSDMD-FL or p3×Flag-C-pGSDMD-1-279aa. At 24 h after transfection, the supernatants were collected and analyzed for LDH levels. (H) The modeled pGSDMD-FL, pGSDMD-N, and pGSDMD-C structures. The analyses were performed by one-way ANOVA with Tukey’s multiple-comparison test (n.s., *P > *0.05; ***, *P < *0.001).

We next investigated whether pGSDMD could induce pyroptosis. Figure 2A shows that cotransfection with plasmids encoding porcine Caspase-1 (pCaspase-1) and pGSDMD significantly increased LDH release in HEK293T cells. To further confirm the results, the cells were analyzed with fluorescence microscopy and flow cytometry (Fig. S3A and B). Both showed that cotransfection with pCaspase-1 and pGSDMD led to increased cell death. The cells were also collected to detect pCaspase-1-mediated cleavage of pGSDMD by immunoblotting. As shown in Fig. 2B, pCaspase-1 could cleave pGSDMD to generate an N-terminal (about 30 kDa) and a C-terminal (about 20 kDa) fragment.

10.1128/mBio.02739-21.3 FIG S3 HEK293T cells were mock transfected or transfected with plasmids as shown. At 24 h after transfection, the cells were processed and stained with PI and then analyzed with fluorescence microscopy (A) and flow cytometry (B). Download FIG S3, JPG file, 1.3 MB. Copyright © 2022 Shi et al. 2022 Shi et al. https://creativecommons.org/licenses/by/4.0/ This content is distributed under the terms of the Creative Commons Attribution 4.0 International license.

It has been reported that human and murine caspases 1 cleave hGSDMD/mGSDMD at the D275-G276 (human) and D276-G277 (murine) junction, respectively, to produce two fragments, p30 and p20 (29). We found that there were two similar-sized fragments about 30 kDa and 20 kDa from cell samples cotransfected with pCaspase-1 and pGSDMD (Fig. 2B). Based on the cleavage site peptide preference of caspase-1 (WEHD/YVHD/FESD) (29–33), the D254-G255 and D279-G280 pairs of pGSDMD were tested as the potential cleaved sites for pCaspase-1. Wild-type pGSDMD, the D254A mutant, or the D279A mutant were cotransfected with pCaspase-1, followed by LDH release and propidium iodide (PI) staining assays. Figure 2C and D shows that D279 mutation resulted in significantly decreased pyroptosis while D254 mutation did not, suggesting that pCaspase-1 cleaved pGSDMD at residue D279-G280. Immunoblotting further confirmed that the wild-type pGSDMD and the D254A mutant were cleaved by pCaspase-1, while the D279A mutant was resistant to the cleavage (Fig. 2E and F). To further validate the results, plasmids encoding pGSDMD_1–279_ were transfected into HEK293T cells. The LDH release assay showed that pGSDMD_1–279_ alone induced pyroptosis (Fig. 2G). Thus, the above-described results suggest that pGSDMD is cleaved by pCaspase-1 at residue D279-G280 and then generates an N-terminal fragment (pGSDMD-p30) to induce pyroptosis (Fig. 2H).

### L295/Y378/A382 are the key sites for pGSDMD autoinhibition.

**FIG 3:**
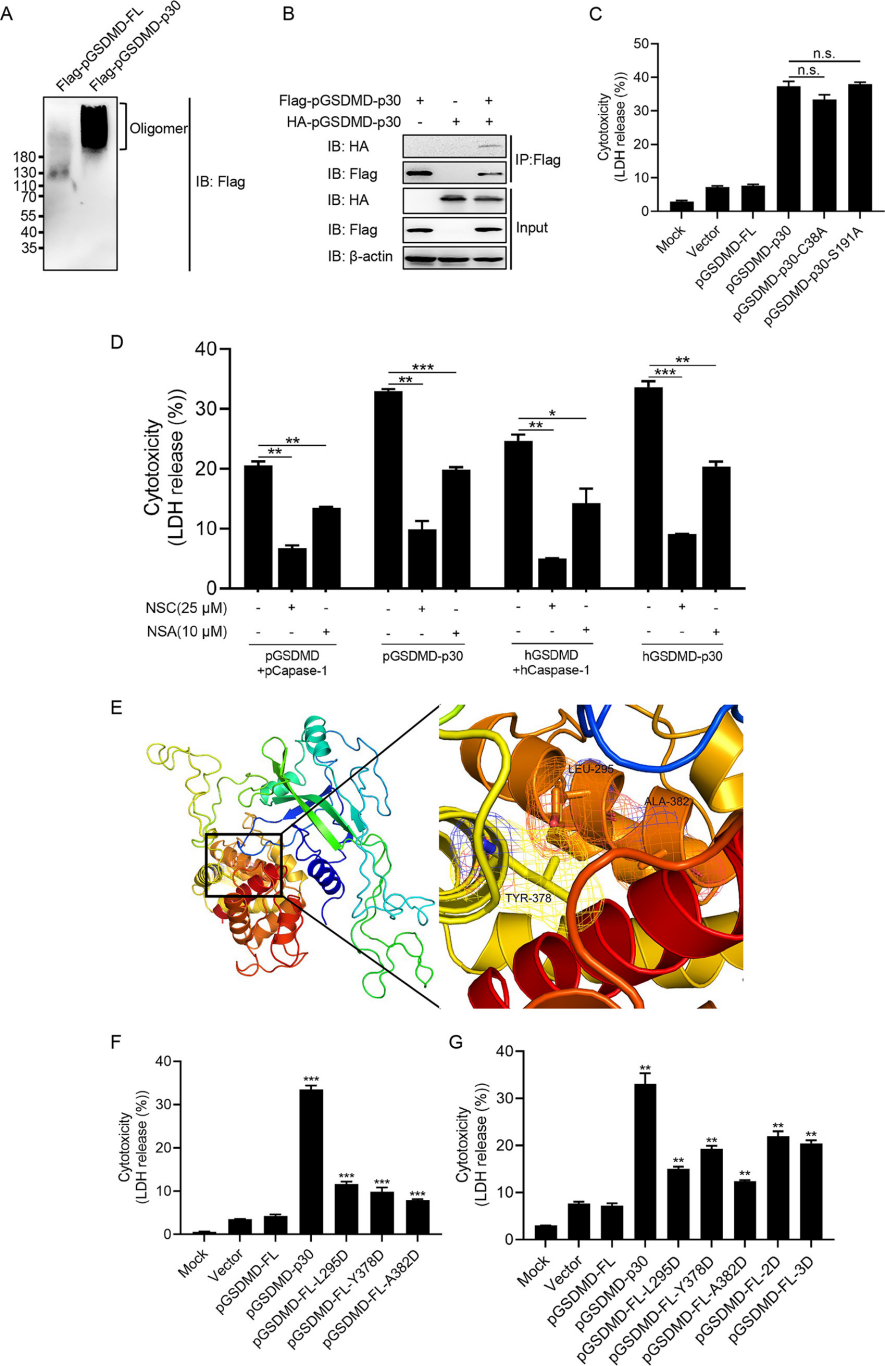
L295/Y378/A382 are the key sites for pGSDMD autoinhibition. (A) HEK293T cells were transfected with plasmids encoding Flag-pGSDMD-FL or Flag-pGSDMD-p30 for 24 h and then lysed under nonreducing conditions and resolved on a native gel. (B) HEK293T cells were transfected with plasmids encoding Flag-pGSDMD-p30 or HA-pGSDMD-p30 or cotransfected with these two plasmids for 24 h, followed by co-IP with anti-Flag binding beads and an immunoblot analysis. (C) HEK293T cells were transfected with plasmids encoding pGSDMD-FL, pGSDMD-p30, or its point mutants. At 24 h after transfection, the supernatants were collected and analyzed for LDH levels. (D) HEK293T cells were transfected with plasmids encoding pGSDMD-FL, pCaspase-1, pGSDMD-p30, hGSDMD-FL, hCaspase-1, and hGSDMD-p30. Meanwhile, cells were mock treated or treated with NSC (final concentration of 25 μM) or NSA (final concentration of 10 μM). At 24 h after transfection, the supernatants were collected and analyzed for LDH levels. (E) The structure of modeled pGSDMD-FL and enlarged view of the boxed area. (F and G) HEK293T cells were transfected with plasmids encoding pGSDMD-p30, pGSDMD-FL, or its point mutants. At 24 h after transfection, the supernatants were collected and analyzed for LDH levels. The analyses were performed by one-way ANOVA with Tukey’s multiple-comparison test (n.s., *P > *0.05; *, *P < *0.05; **, *P < *0.01; ***, *P < *0.001).

It has been reported that the residues C38/C39 and C191/C192 (human/murine) are essential for oligomerization of the GSDMD N terminus (18, 34). We found that pGSDMD-p30 did oligomerize (Fig. 3A and B). Based on the multiple-sequence alignment of GSDMDs (Fig. S1), residues C38 and S191 were tested as the potential key sites for pGSDMD-p30 to oligomerize. The HEK293T cells transfected with the mutants C38A and S191A still showed significant pyroptotic death, shown as increased LDH release (Fig. 3C). Specific inhibitors, known for inhibition of human GSDMD-p30 oligomerization, NSC (tetraethylthiuram disulfide) and NSA (necrosulfonamide) (34, 35), could also inhibit pGSDMD-p30-induced pyroptosis (Fig. 3D). It should be noted that NSC and NSA may directly inhibit pGSDMD-p30 oligomerization or do so through a different manner than hGSDMD-p30 to suppress pGSDMD-p30-mediated pyroptosis.

Earlier reports demonstrated that the full length of hGSDMD has an autoinhibitory structure in which the GSDMD C terminus inhibits the pore-forming activity of GSDMD-N. L290, Y373, and A377 of hGSDMD are key residues for hGSDMD autoinhibition (20, 34). Based on the multiple-sequence alignment, the equivalent residues in pGSDMD are L295, Y378, and A382 and might be involved in forming a pocket associated with the pGSDMD N terminus according to the homology modeling (Fig. 3E). Thus, the three residues were tested as the potential sites in pGSDMD. These residues were individually mutated to D (pGSDMD-FL-L295D/pGSDMD-FL-Y378D/pGSDMD-FL-A382D), both L295 and Y373 to D (pGSDMD-FL-2D), and all three residues to D (pGSDMD-FL-3D). The mutants were transfected into HEK293T cells for analysis of LDH release. All the mutants were able to induce pyroptosis but to a lesser extent than pGSDMD-p30 (Fig. 3F). However, there seemed to be no additive effect of double or triple mutants in LDH release (Fig. 3G). The aforementioned results suggest that L295, Y378, and A382 are the critical sites for autoinhibitory structure of the full length of pGSDMD.

### PEDV Nsp5 associates with and cleaves pGSDMD.

**FIG 4:**
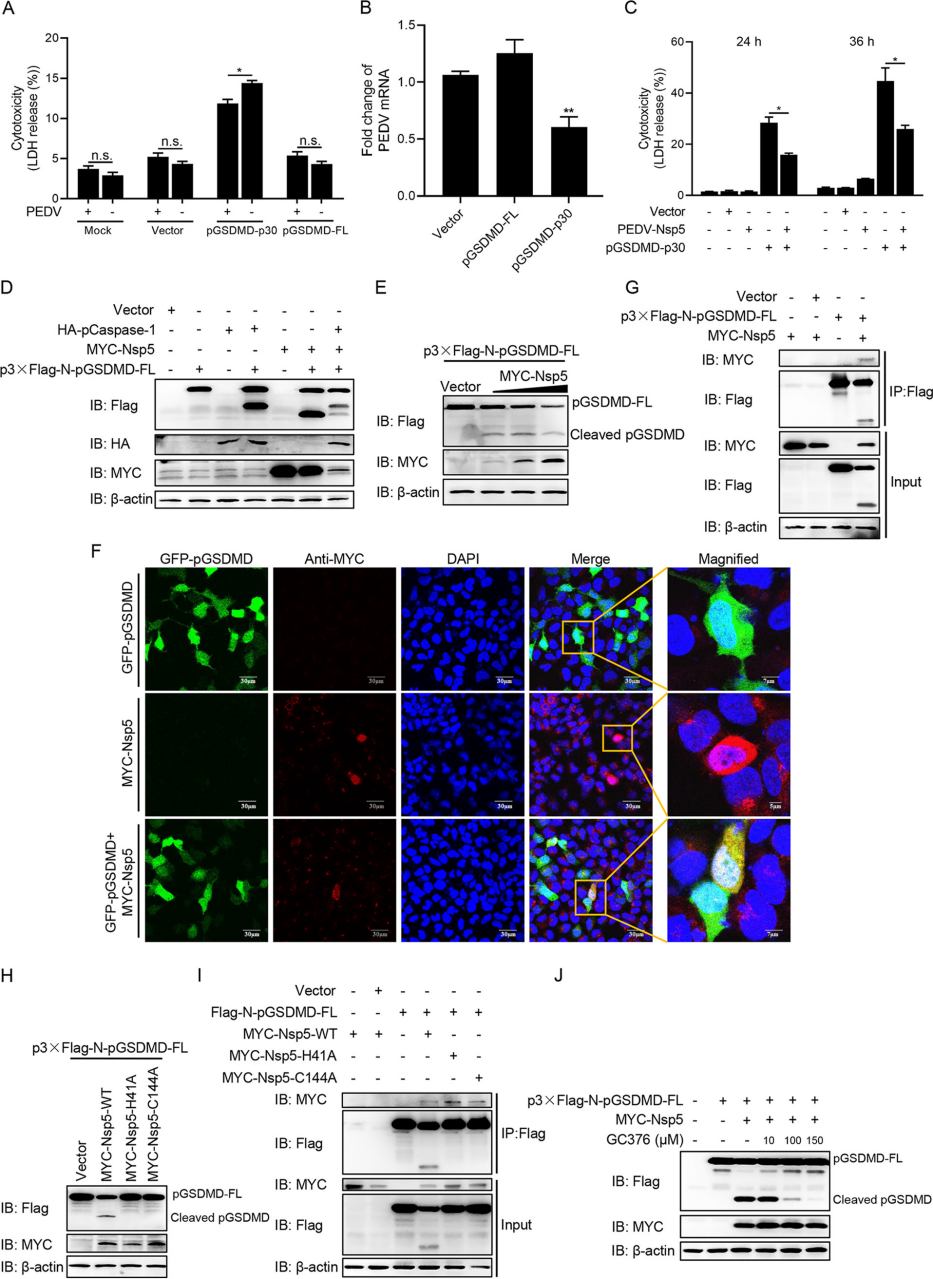
PEDV Nsp5 associates with and cleaves pGSDMD. (A) Vero cells were transfected with plasmids encoding pGSDMD-p30 or pGSDMD-FL. At 4 h after transfection, the cells were mock infected or infected with PEDV at an MOI of 0.1. After 36 h, the supernatants were collected and analyzed for LDH levels. (B) Vero cells were transfected with plasmids encoding pGSDMD-p30 or pGSDMD-FL. At 24 h after transfection, the cells were infected with PEDV at an MOI of 0.5. After 24 h, total RNA was extracted and the viral RNA levels of PEDV were evaluated by quantitative real-time PCR using SYBR green. Data were expressed as fold change of the PEDV mRNA level relative to that of the control vector. (C) HEK293T cells were transfected with plasmids encoding PEDV-Nsp5 or pGSDMD-p30 or cotransfected with these two plasmids. At 24 h and 36 h after transfection, the supernatants were collected and analyzed for LDH levels. (D) HEK293T cells were transfected with plasmids as shown. At 24 h after transfection, the cells were processed for immunoblotting. (E) HEK293T cells were cotransfected with plasmids encoding p3×Flag-N-pGSDMD-FL and various doses of MYC-Nsp5. After 24 h, cells were lysed for immunoblotting. (F) HEK293T cells were cotransfected with plasmids encoding GFP-pGSDMD and MYC-Nsp5 for 24 h, and then MYC-Nsp5 cells were labeled with specific primary antibodies and secondary antibodies (red). Cell nuclei were stained with DAPI (blue). The fluorescent signals were observed with confocal immunofluorescence microscopy. HEK293T cells were transfected with plasmids encoding GFP-pGSDMD or MYC-Nsp5 as a control. (G) HEK293T cells were transfected with plasmids as shown for 24 h, followed by co-IP with anti-Flag binding beads and an immunoblot analysis. (H) HEK293T cells were cotransfected with plasmids encoding p3×Flag-N-pGSDMD-FL and wild-type PEDV Nsp5 or its protease-defective mutants (H41A and C144A). After 24 h, cells were lysed for immunoblotting. (I) HEK293T cells were transfected with plasmids as shown, followed by co-IP with anti-Flag binding beads and an immunoblot analysis. (J) HEK293T cells were transfected with the plasmids as shown, and 6 h after transfection, cells were mock treated or treated with different doses of GC376 (final concentration of 10, 100, or 150 μM). At 24 h after transfection, cells were lysed for immunoblotting. The analyses were performed by one-way ANOVA with Tukey’s multiple-comparison test. The experiment shown in panel A was carried out by Student's *t* test (n.s., *P > *0.05; *, *P < *0.05; **, *P < *0.01).

To investigate the relationship between PEDV infection and pyroptosis, Vero cells were transfected with plasmids encoding full-length pGSDMD (pGSDMD-FL) or the GSDMD N terminus (pGSDMD-p30) and then infected with PEDV. The LDH release assay showed that PEDV infection had an inhibitory effect on pyroptosis induced by pGSDMD-p30 (Fig. 4A), while its replication was significantly inhibited by pGSDMD-p30 expression (Fig. 4B). These results suggest that there is a reciprocal regulation between PEDV replication and pGSDMD-p30-mediated pyroptosis.

Nonstructural protein 5 (Nsp5), the 3C-like protease, has been reported to cleave a number of host proteins to suppress antiviral host responses (5, 8, 9, 13). We speculated that PEDV Nsp5 could cleave pGSDMD to suppress pyroptosis. As shown in Fig. 4C, PEDV Nsp5 inhibited pyroptosis induced by pGSDMD-p30. For further validation, HEK293T cells were transfected with plasmids as indicated in Fig. 4D for immunoblotting. There was a faster-migrating protein band (about 25 kDa) in the samples cotransfected with PEDV Nsp5 and p3×Flag-N-pGSDMD-FL (Fig. 4D, lane 6), and there were two cleavage protein bands, of 35 kDa (p30) and 25 kDa, in the samples cotransfected with hemagglutinin (HA)-pCaspase-1, PEDV Nsp5, and p3×Flag-N-pGSDMD-FL (Fig. 4D, lane 7). These results imply that pGSDMD was a cleaved target of PEDV Nsp5. It is known that D87 of hGSDMD is the active caspase-3 cleavage site (36), and next we transfected HEK293T cells with PEDV Nsp5 and p3xFlag-N-pGSDMD-FL or its mutant, p3xFlag-N-pGSDMD-FL-D87A. As shown in Fig. S4A, D87A mutant did not impair Nsp5 cleavage on pGSDMD. Cleavage of pGSDMD increased progressively in an Nsp5 dose-dependent manner (Fig. 4E, Fig. S4B). Indirect immunofluorescence showed that pGSDMD and Nsp5 colocalized in the cytoplasm (Fig. 4F). The coimmunoprecipitation (co-IP) experiments also demonstrated that PEDV Nsp5 interacted with and cleaved pGSDMD (Fig. 4G).

10.1128/mBio.02739-21.4 FIG S4 (A) HEK293T cells were cotransfected with plasmids encoding PEDV-Nsp5 and p3×Flag-N-pGSDMD-FL or its mutant, p3×Flag-N-pGSDMD-FL-D87A. After 24 h, cells were lysed for immunoblotting. (B) HEK293T cells were cotransfected with plasmids encoding p3×Flag-C-pGSDMD-FL and various doses of MYC-Nsp5. After 24 h, cells were lysed for immunoblotting. Download FIG S4, JPG file, 0.2 MB. Copyright © 2022 Shi et al. 2022 Shi et al. https://creativecommons.org/licenses/by/4.0/ This content is distributed under the terms of the Creative Commons Attribution 4.0 International license.

It is known that H41 and C144 of PEDV Nsp5 are critical for its protease activity (6, 37–39). To further investigate whether PEDV Nsp5 cleaves pGSDMD by means of its protease activity, two Nsp5 mutants, H41A and C144A, were cotransfected with p3×Flag-N-pGSDMD-FL into HEK293T cells. As shown in Fig. 4H, wild-type Nsp5 cleaved pGSDMD successfully, while the two mutants did not. Nevertheless, co-IP experiments showed that the Nsp5 mutants, having lost the protease activity, were still able to interact with pGSDMD (Fig. 4I). Hence, the protease activity of PEDV Nsp5 is essential for pGSDMD cleavage. In addition, the cleavage of pGSDMD by PEDV-Nsp5 was significantly inhibited by GC376, an inhibitor of 3C-like protease (40–43), in a dose-dependent manner (Fig. 4J).

### PEDV Nsp5 cleaves pGSDMD at residue Q193-G194.

**FIG 5:**
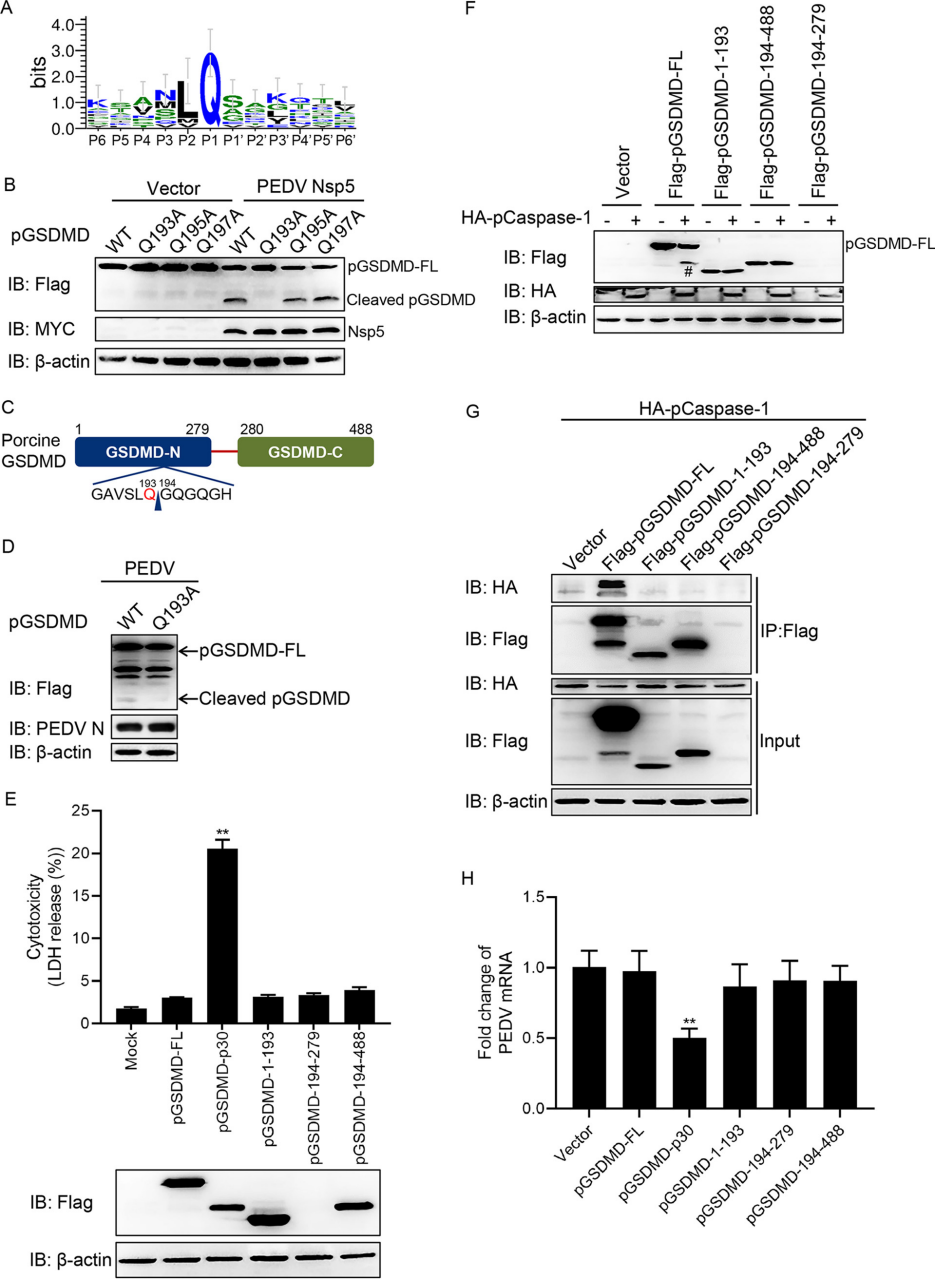
PEDV Nsp5 cleaves pGSDMD at residue Q193-G194. (A) Logo analysis of the cleavage site predicted from the polyprotein cleavage of PEDV Nsp5. (B) HEK293T cells were cotransfected with plasmids encoding MYC-Nsp5 and p3×Flag-N-pGSDMD-FL or its mutants, p3×Flag-N-pGSDMD-FL-Q193A, p3×Flag-N-pGSDMD-FL-Q195A, and p3×Flag-N-pGSDMD-FL-Q197A. Cells were then lysed after 24 h and evaluated by immunoblotting. (C) Cartoon diagram of pGSDMD structure and the cleavage site by PEDV Nsp5. (D) Vero cells were transfected with plasmids encoding p3×Flag-N-pGSDMD-FL or its mutant, p3×Flag-N-pGSDMD-FL-Q193A. At 12 h after transfection, cells were infected with PEDV at an MOI of 1. Cells were then lysed after 16 h and evaluated by immunoblotting. (E) HEK293T cells were transfected with plasmids encoding pGSDMD-FL, pGSDMD-p30, pGSDMD-1-193, pGSDMD-194-279, or pGSDMD-194-279 with a Flag tag. After 24 h, the supernatants were collected and analyzed for LDH levels, and the cells were then processed for immunoblotting. (F) HEK293T cells were transfected with the plasmids as shown. After 24 h, the cells were then processed for immunoblotting (#, pGSDMD-p30). (G) HEK293T cells were cotransfected with plasmids encoding HA-pCaspase-1 and Flag-pGSDMD-FL, Flag-pGSDMD-1-193, Flag-pGSDMD-194-488, or Flag-pGSDMD-194-279, followed by co-IP with anti-Flag binding beads and an immunoblot analysis. (H) Vero cells were transfected with plasmids encoding pGSDMD or its variants as indicated. At 24 h after transfection, cells were infected with PEDV at an MOI of 0.5. After 24 h, total RNA was extracted, and the viral RNA levels of PEDV were evaluated by quantitative real-time PCR using SYBR green. The analyses were performed by one-way ANOVA with Tukey’s multiple-comparison test (**, *P < *0.01).

Logo analysis of the cleavage site predicted from the polyprotein cleavage of PEDV Nsp5 is shown in Fig. 5A. Based on the substrate preference of Nsp5 and the sizes of the cleaved bands, the Q193-G194, Q195-G196, and Q197-G198 pairs were tested as the potential cleaved sites (44, 45). Therefore, these three mutants, pGSDMD-Q193A, pGSDMD-Q195A, and pGSDMD-Q197A, were cotransfected with PEDV Nsp5. As shown in Fig. 5B, pGSDMD-Q193A was resistant to PEDV Nsp5 cleavage, while pGSDMD-Q195A and pGSDMD-Q197A were not, suggesting that PEDV Nsp5 cleaves pGSDMD at the residue Q193-G194 junction (Fig. 5C). Further study demonstrated that PEDV infection also led to pGSDMD cleavage at residue Q193 (Fig. 5D).

PEDV Nsp5 cleaves pGSDMD to generate pGSDMD_1–193_ and pGSDMD_194–488_, and pCaspase-1 cleaves pGSDMD at residue D279. These cleaved fragments, pGSDMD_1–193_, pGSDMD_194–279_, and pGSDMD_194–488_, did not induce pyroptosis (Fig. 5E). Since the protein band of pGSDMD_194–279_ was too small to be visualized, we subsequently cloned them into GFP-tagged vectors and then transfected into HEK293T cells. We found that these three truncated mutants could not induce pyroptosis (Fig. S5). Next, we further examined whether pCaspase-1 could associate with and cleave these three truncated mutants. As shown in Fig. 5F and G, pCaspase-1 could associate with and cleave the full length of pGSDMD but had no interaction with pGSDMD_1–193_, pGSDMD_194–279_, and pGSDMD_194–488_.

10.1128/mBio.02739-21.5 FIG S5 HEK293T cells were mock transfected or transfected with the plasmids encoding pGSDMD-FL, pGSDMD-p30, pGSDMD-1-193, pGSDMD-194-279, and pGSDMD-194-488 with a GFP tag. After 24 h, the supernatants were collected and analyzed for LDH levels, and the cells were analyzed with fluorescence microscopy. The analyses were performed by one-way ANOVA with Tukey’s multiple-comparison test (*, *P < *0.05). Download FIG S5, JPG file, 0.4 MB. Copyright © 2022 Shi et al. 2022 Shi et al. https://creativecommons.org/licenses/by/4.0/ This content is distributed under the terms of the Creative Commons Attribution 4.0 International license.

As described above, pyroptosis induced by pGSDMD-p30 had an inhibitory effect on PEDV replication. We next investigated whether PEDV Nsp5-mediated cleavage products of pGSDMD would affect PEDV replication. Vero cells were transfected with plasmids encoding pGSDMD-FL, pGSDMD-p30, pGSDMD_1–193_, pGSDMD_194–279_, or pGSDMD_194–488_. At 24 h after transfection, cells were infected with PEDV for another 24 h, and then PEDV replication was evaluated by real-time quantitative PCR (RT-qPCR). As shown in Fig. 5H, there were no statistical differences of viral mRNA among vector, pGSDMD-FL, pGSDMD_1–193_, pGSDMD_194–279_, and pGSDMD_194–488_, indicating that the cleaved fragments had no inhibitory effect on PEDV replication.

### Amino acids R238, T239, and F240 are key sites for pGSDMD-p30 to induce pyroptosis.

**FIG 6:**
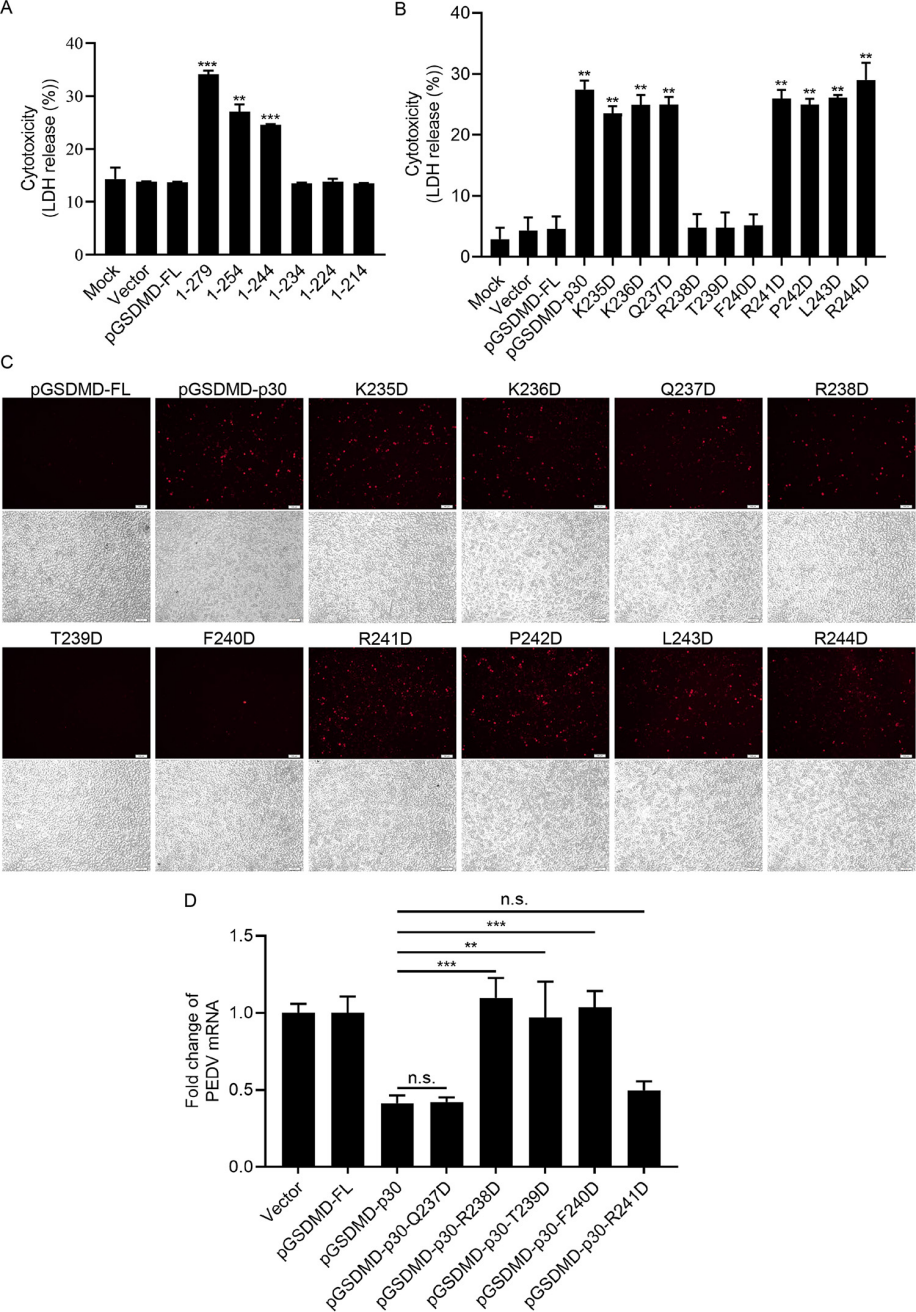
Amino acids R238, T239, and F240 are key sites for pGSDMD-p30 to induce pyroptosis. (A) HEK293T cells were transfected with plasmids encoding pGSDMD-FL or its variants. After 24 h, the supernatants were collected and analyzed for LDH levels. (B and C) HEK293T cells were transfected with plasmids encoding pGSDMD-p30 or its point mutants. After 24 h, the supernatants were collected and analyzed for LDH levels (B), and the cells were also dyed with PI (C). (D) Vero cells were transfected with the plasmids encoding pGSDMD-FL, pGSDMD-p30, or its point mutants (pGSDMD-p30-Q237D, pGSDMD-p30-R238D, pGSDMD-p30-T239D, pGSDMD-p30-F240D, and pGSDMD-p30-R241D). At 24 h after transfection, cells were infected with PEDV at an MOI of 0.5. After 24 h, total RNA was extracted, and the viral RNA levels of PEDV were evaluated by quantitative real-time PCR using SYBR green. The analyses were performed by one-way ANOVA with Tukey’s multiple-comparison test (**, *P* < 0.01; ***, *P* < 0.001).

It has been shown that pGSDMD_1–279_ (pGSDMD-p30) can induce pyroptosis, while pGSDMD_1–193_ cannot. Based on this, we conjectured that the active motif of pGSDMD to induce pyroptosis is located at the amino acids between 193 and 279. Thus, we constructed a series of pGSDMD truncated mutants encoding pGSDMD_1–254_, pGSDMD_1–244_, pGSDMD_1–234_, pGSDMD_1–224_, and pGSDMD_1–214_ and transfected them into HEK293T cells. As shown in Fig. 6A and Fig. S6, pGSDMD_1–279_, pGSDMD_1–254_, and pGSDMD_1–244_ induced pyroptosis while pGSDMD_1–234_, pGSDMD_1–224_, and pGSDMD_1–214_ did not, indicating that the key sites located between amino acids 234 and 244. Hence, the amino acids between 234 and 244 were replaced by D, and these point mutant plasmids were transfected into HEK293T cells. The results showed that all of the point mutants, except T239D and F240D, induced pyroptosis, suggesting that T239 and F240 are the essential sites for pGSDMD-p30 to induce pyroptosis (Fig. 6B). The results were further proved by PI staining (Fig. 6C). Notably, the point mutant R238D inhibited the LDH release but did not inhibit the intake of PI, indicating that the R238 mutation led to smaller pores on cell membrane than wild-type pGSDMD-p30.

10.1128/mBio.02739-21.6 FIG S6 HEK293T cells were transfected with plasmids encoding pGSDMD-FL or its variants. After 24 h, the cells were dyed with PI and analyzed with fluorescence microscopy. Download FIG S6, JPG file, 0.5 MB. Copyright © 2022 Shi et al. 2022 Shi et al. https://creativecommons.org/licenses/by/4.0/ This content is distributed under the terms of the Creative Commons Attribution 4.0 International license.

To further investigate the effects of R238, T239D, and F240D on viral replication, the Q237D/R238D/T239D/F240D/R241D mutants were transfected into Vero cells and then infected with PEDV. Figure 6D shows that Q237D/R241D mutants could still inhibit PEDV replication similar to their parental peptide pGSDMD-p30 while R238D/T239D/F240D did not, further confirming that inhibition of pGSDMD-p30-induced pyroptosis is important for PEDV replication.

### GSDMD is a common substrate of different coronaviruses Nsp5.

**FIG 7:**
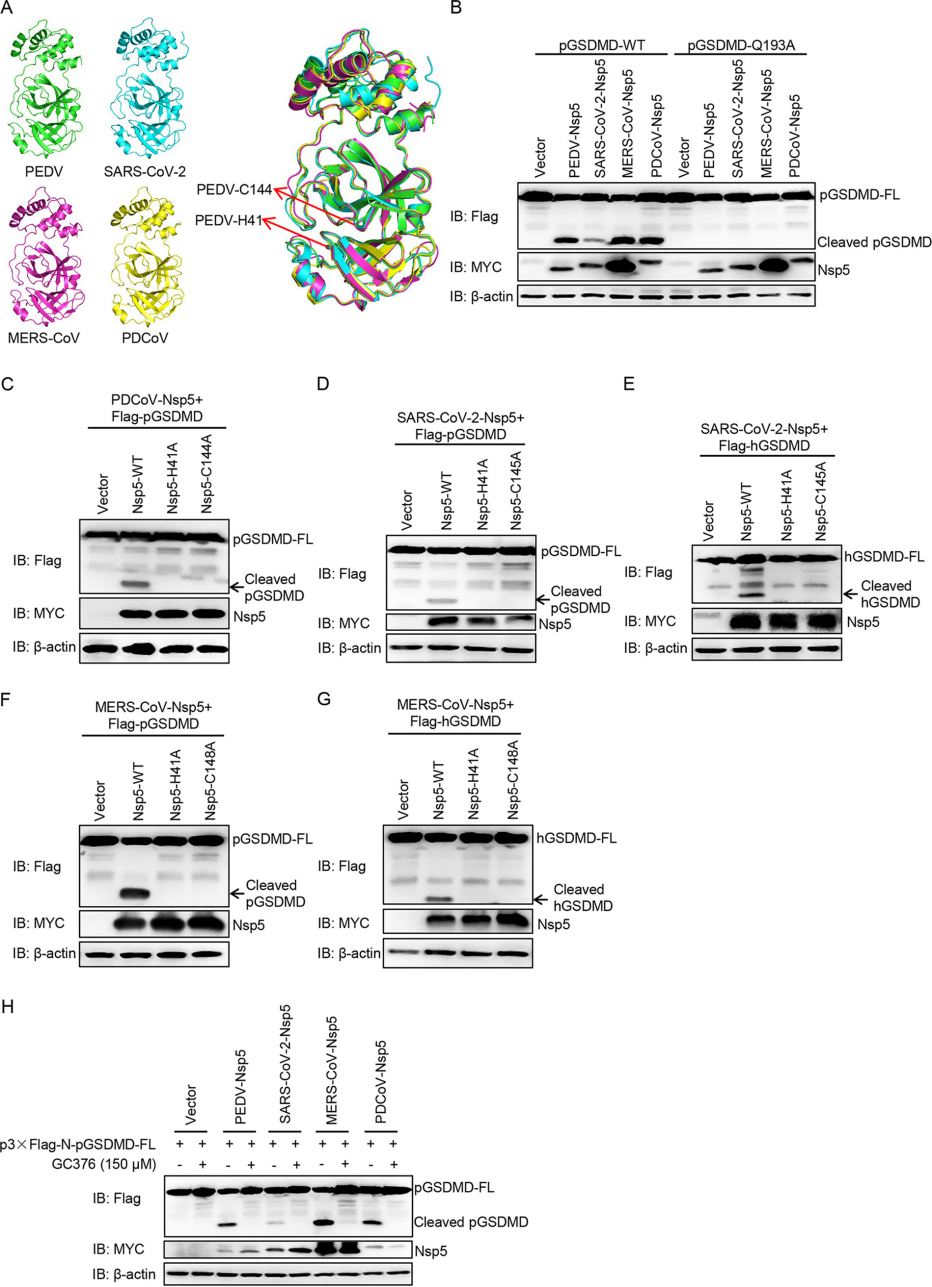
GSDMD is a common substrate of different coronaviruses Nsp5. (A) Structure alignment of CoVs Nsp5. Red arrows indicate conserved enzymatic proteolysis residues His41 and Cys144. The three-dimensional structures were derived from the Protein Data Bank with the following accession numbers: PEDV, 4XFQ; SARS-CoV-2, 7BUY; MERS-CoV, 5WKK; PDCoV, 6JIJ. (B) HEK293T cells were cotransfected with plasmids encoding p3×Flag-N-pGSDMD-FL or p3×Flag-N-pGSDMD-FL-Q193A and Nsp5 encoded by PEDV, PDCoV, SARS-CoV-2 and MERS-CoV. After 24 h, cells were lysed and detected by immunoblotting. (C) HEK293T cells were cotransfected with plasmids encoding pGSDMD and wild-type PDCoV Nsp5 or its protease-defective mutants (H41A and C144A). After 24 h, cells were lysed for immunoblotting. (D and E) HEK293T cells were cotransfected with plasmids encoding pGSDMD and wild-type SARS-CoV-2 Nsp5 or its protease-defective mutants (H41A and C145A), hGSDMD, and wild-type SARS-CoV-2 Nsp5 or its protease-defective mutants (H41A and C145A). After 24 h, cells were lysed for immunoblotting. (F and G) HEK293T cells were cotransfected with plasmids encoding pGSDMD and wild-type MERS-CoV Nsp5 or its protease-defective mutants (H41A and C148A), hGSDMD, and wild-type MERS-CoV Nsp5 or its protease-defective mutants (H41A and C148A). After 24 h, cells were lysed for immunoblotting. (H) HEK293T cells were cotransfected with plasmids encoding p3×Flag-N-pGSDMD-FL and Nsp5 encoded by PEDV, PDCoV, SARS-CoV-2, and MERS-CoV. At 6 h after transfection, cells were mock treated or treated with GC376 (final concentration of 150 μM). After 24 h, cells were lysed for immunoblotting.

Next, we tested whether Nsp5 in other CoVs could cleave GSDMD. Multiple-sequence alignment showed that Nsp5 of PDCoV, SARS-CoV-2, and MERS-CoV are highly similar to PEDV Nsp5 (Fig. S7), especially in their catalytic domains (Fig. 7A). Thus, the Nsp5 of PDCoV, SARS-CoV-2, and MERS-CoV were cotransfected with pGSDMD, and the results showed that all these Nsp5 proteins could cleave pGSDMD at residue Q193-G194 (Fig. 7B). It has been reported that histidine (His) and cysteine (Cys) residues of CoVs Nsp5 form a catalytic dyad, and any mutation in the catalytic sites can disrupt its protease activity (6, 46, 47). In addition, previous studies have demonstrated that PDCoV Nsp5 H41A/C144A, SARS-CoV-2 Nsp5 H41A/C145A, and MERS-CoV Nsp5 H41A/C148A do not show protease activity (37, 48–50). As shown in Fig. 7C, PDCoV Nsp5 cleaved pGSDMD, while its protease-dead mutants did not. Likewise, the wild-type Nsp5 of SARS-CoV-2 cleaved both pGSDMD (Fig. 7D) and hGSDMD (Fig. 7E), while its mutants did not. Similar results were observed for cleavage of pGSDMD and hGSDMD by MERS-CoV Nsp5 (Fig. 7F and G). In addition, the inhibitor for coronavirus Nsp5, GC376, significantly decreased pGSDMD cleavage in all tested coronavirus Nsp5 proteins (Fig. 7H). The results described above suggest that Nsp5 from SARS-CoV-2, MERS-CoV, PDCoV, and PEDV can cleave pGSDMD at the Q193-G194 junction, and Nsp5 from SARS-CoV-2 and MERS-CoV also cleaves hGSDMD.

10.1128/mBio.02739-21.7 FIG S7 Alignment of the amino acid sequence of Nsp5 of PEDV with Nsp5 of PDCoV (GenBank accession no. AKQ63081.1), SARS-CoV-2 (GenBank accession no. NC_045512), and MERS-CoV (GenBank accession no. NC_038294). Download FIG S7, JPG file, 1.0 MB. Copyright © 2022 Shi et al. 2022 Shi et al. https://creativecommons.org/licenses/by/4.0/ This content is distributed under the terms of the Creative Commons Attribution 4.0 International license.

To further validate this conclusion, we analyzed the peptides GAVSLQ(193)↓GQGQGH (pGSDMD; arrow represents cleavage site) and Nsp5 of PEDV (Fig. S8A), SARS-CoV-2 (Fig. S8B), MERS-CoV (Fig. S8C), and PDCoV (Fig. S8D) by homology modeling (47, 51). As shown in Fig. S8, the residues of pGSDMD comfortably fit in the Nsp5 pockets of different CoVs, suggesting a strong interaction between them.

10.1128/mBio.02739-21.8 FIG S8 Homology modeling of Nsp5 of different CoVs with the cleaved pGSDMD peptide substrate. The molding structures of Nsp5 from PEDV (PDB accession number 4XFQ) (A), SARS-CoV-2 (PDB accession number 7BUY) (B), MERS-CoV (PDB accession number 5WKK) (C), and PDCoV (PDV accession number 6JIJ) (D) combined with the cleaved pGSDMD peptide substrate GAVSLQ(193)↓GQGQGH (downward arrows indicates cleavage sites) were analyzed using PyMOL software. Download FIG S8, JPG file, 0.7 MB. Copyright © 2022 Shi et al. 2022 Shi et al. https://creativecommons.org/licenses/by/4.0/ This content is distributed under the terms of the Creative Commons Attribution 4.0 International license.

## DISCUSSION

**FIG 8:**
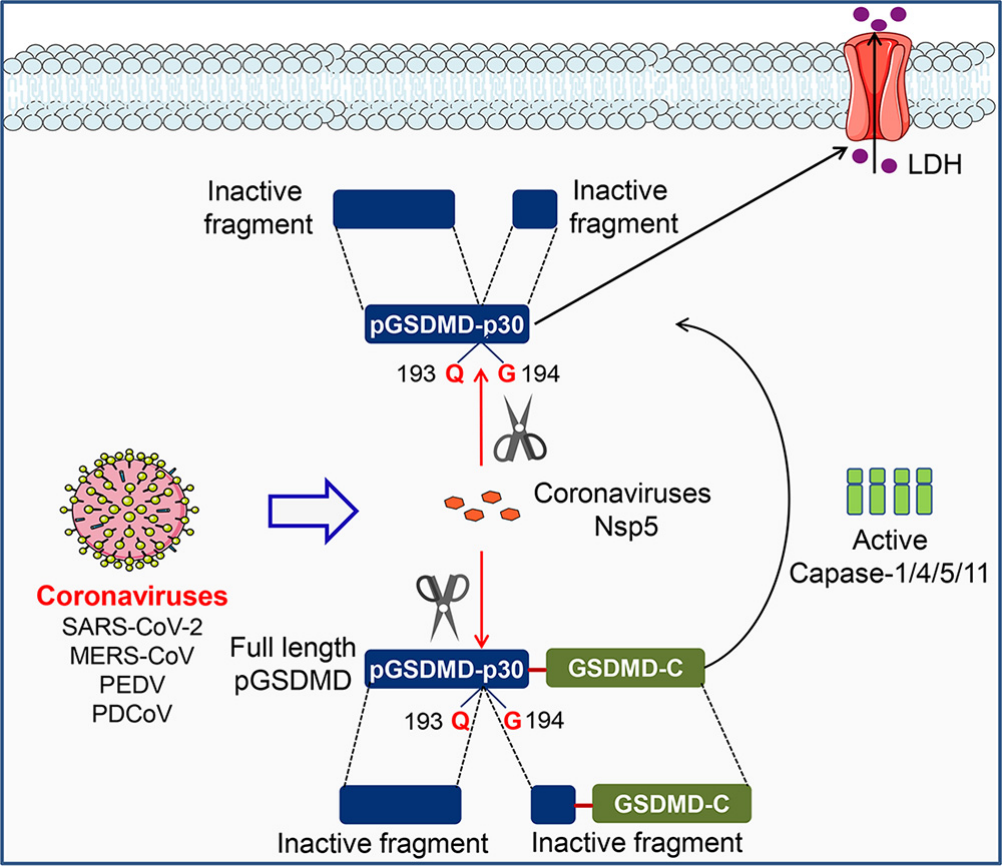
Mechanistic diagram illustrating antagonization of GSDMD-mediated pyroptosis by Nsp5 from coronaviruses.

Although considerable progress has been made in CoV research, knowledge gaps still exist with respect to the host innate immune responses against CoV infection. Here, we used PEDV as a model of CoV to illustrate the relationship between PEDV replication and pyroptosis (Fig. 8). We have demonstrated that pGSDMD plays a protective role against PEDV infection. Upon infection, PEDV deploys Nsp5 to cleave pGSDMD at the Q193-G194 junction to produce two fragments that were inactive in pyroptosis induction, thus favoring its replication. Nsp5 from SARS-CoV-2, MERS-CoV, and PDCoV can also cleave hGSDMD and pGSDMD. Thus, our results demonstrate that GSDMD may be an appealing target for the design of anticoronavirus therapies.

Recent studies have identified that human/murine GSDMD is a direct substrate of caspase-1/4/5/11 and serves as the executioner for pyroptosis. However, the amino acid sequence and molecular characterization of porcine GSDMD have not been illustrated. To investigate the role of pGSDMD-mediated pyroptosis in PEDV infection, we first clarified that porcine GSDMD, 488 amino acids (aa) in length, can be cleaved by pCaspase-1 at D279 to produce pGSDMD-p30, an active peptide, to induce pyroptosis. Early site-directed mutagenesis studies revealed that C38/C39 and C191/C192 (human/murine) mutations impaired hGSDMD-p30/mGSDMD-p30 oligomerization (19, 34). However, our results indicate that mutation of pGSDMD-p30 C38 or S191 (corresponding to human C38 and C191) had no inhibitory effect on pyroptosis. Interestingly, inhibitors of hGSDMD-p30 oligomerization could also suppress pGSDMD-p30-induced pyroptosis. Future work is needed to clarify whether NSC and NSA directly inhibit pGSDMD-p30 oligomerization or do so through a different way than hGSDMD-p30 to suppress pGSDMD-p30-induced pyroptosis.

It has been reported that the 3C-like proteases of CoVs are involved in evading host innate immune responses (8–10). Our present study first demonstrated that CoVs Nsp5 can cleave and inactivate pGSDMD. Thus, GSDMD represents a novel target of CoV Nsp5. PEDV Nsp5 not only interacts with and cleaves the full length of pGSDMD but also inhibits pGSDMD-p30-induced pyroptosis by cleaving pGSDMD-p30. Conversely, protease-dead mutants of the four tested CoV Nsp5 proteins are unable to cleave human and porcine GSDMD. Thus, these results suggest a reciprocal regulation between CoV Nsp5 and pyroptosis.

It is known that active caspase-3 can also block pyroptosis by cleaving hGSDMD at D87 (36). PEDV infection can induce caspase-3 activation and subsequent apoptosis in various host cells (52–54), and the S1 protein from SARS-CoV and MERS-CoV also induces Vero-E6 cell apoptosis (54). Therefore, it is reasonable to infer that PEDV might use two ways to inhibit host cell pyroptosis. Indirectly, PEDV utilizes host cell proteins, such as caspase-3, by activation to prevent pyroptosis by cutting GSDMD at the D87 site. Directly, PEDV uses its own protease, Nsp5, to cleave GSDMD at the Q193 site to inactivate the pyroptotic response. Our present study focused on the direct relationship between coronavirus Nsp5 and GSDMD-mediated pyroptosis.

It is noteworthy that PEDV Nsp5 cleaves pGSDMD at the Q193-G194 junction. Our results suggest that amino acids R238, T239, and F240 within pGSDMD-p30 are critical for pyroptosis. Upon cleavage by PEDV Nsp5, the truncated N-terminal fragment without R238, T239, and F240 sites failed to induce LDH release and could not inhibit PEDV replication. Interestingly, a newly published study demonstrated that Zika virus (ZIKV) protease directly cleaved hGSDMD into an N-terminal fragment (1-249) that contains R238, T239, and F240. ZIKV NS2B3 protease cleaves hGSDMD at residue R249 to produce the hGSDMD_1–249_ fragment, which leads to pyroptosis in a caspase-independent manner (55). Consistent with this, a previous study demonstrated that NS5 protein of ZIKA could directly interact with NLRP3 protein and facilitate NLRP3 inflammasome activation (56), which is an upstream event for hGSDMD-p30-mediated pyroptosis. Therefore, viruses use different strategies to evade host immune responses to benefit their replication.

In summary, we used PEDV as a model of coronaviruses to illustrate the reciprocal regulation between CoV infection and pyroptosis. For the first time, we illustrate the molecular mechanism of pGSDMD-mediated pyroptosis and demonstrate that amino acids R238, T239, and F240 within pGSDMD-p30 are critical for pyroptosis. Furthermore, Nsp5 from SARS-CoV-2, MERS-CoV, PDCoV, and PEDV can cleave pGSDMD at the Q193-G194 junction to produce two fragments inactive in pyroptosis induction. The two cleaved fragments do not inhibit PEDV replication. In addition, SARS-CoV-2 Nsp5 and MERS-CoV Nsp5 also cleave hGSDMD. Thus, we provide clear evidence that coronaviruses might utilize Nsp5 to inhibit pyroptosis in favor of their replication. Coincidentally, during our revision process, the latest study demonstrated that SARS-CoV-2 nucleocapsid protein associates with hGSDMD in cells and inhibits hGSDMD cleavage *in vitro* and *in vivo*. SARS-CoV-2 nucleocapsid directly binds the linker region of GSDMD to protect GSDMD tetrapeptide from being cut by caspase-1 (57). This indicates that other proteins of coronaviruses also participate in suppressing GSDMD-mediated pyroptosis. It will be interesting to investigate whether Nsp5 and nucleocapsid protein work together to inhibit GSDMD-mediated pyroptosis during CoV infection. In addition, this study is helpful for researchers to investigate whether other proteases of noncoronaviruses also possess the ability to cleave GSDMD or other gasdermin-mediated pyroptosis to benefit their replication.

## MATERIALS AND METHODS

### Cells and virus.

Vero cells and HEK293T cells were cultured in Dulbecco’s modified Eagle’s medium (DMEM) (HyClone) containing 10% fetal bovine serum (FBS) and 5% penicillin-streptomycin solution (HyClone). IPEC-J2 cells were maintained in DMEM-F12 (HyClone) supplemented with 10% FBS and 5% penicillin-streptomycin solution. When cells seeded in culture plates grew to approximately 60%, they were transfected with plasmids using VigoFect (Vigorous Biotechnology) or Lipo8000 transfection reagent (Beyotime, Shanghai, China) according to the manufacturer’s instructions. Porcine small intestinal crypts were isolated from 3-week-old healthy Yorkshire piglets, and the crypts were seeded in a 48-well plate to culture porcine intestinal enteroids (PIEs) as described in our previous protocol (58). The animal study was reviewed and approved by the Animal Care and Use Committee of Zhejiang University.

The PEDV strain ZJ15XS0101 (GenBank accession no. KX55SO0281) was isolated and stored in our laboratory (59). Vero cells and IPEC-J2 cells grown to approximately 80% to 90% in cell culture plates were infected with PEDV at different multiplicities of infection (MOI) with 4 μg/ml trypsin.

### Plasmids and antibodies.

All eukaryotic expression vectors used in this study were preserved in our laboratory. The porcine GSDMD gene was amplified from cDNA of 3D4/21 cells and cloned into p3×Flag-N vector and p3×Flag-C vector, and the substitution mutants and truncated mutants of GSDMD were also cloned into p3×Flag-N vector and p3×Flag-C vector. The porcine caspase-1 gene was amplified from cDNA of 3D4/21 cells and cloned into pCMV-HA vector. The plasmid encoding PEDV Nsp5 and its substitution mutants were generated by cloning the cDNA sequence of Nsp5 into PRK-MYC vector. The plasmids encoding Nsp5 of SARS-CoV-2, MERS-CoV, and PDCoV were synthesized by Sangon Biotech. Primers used for PCR are listed in Table S1A and B in the supplemental material.

10.1128/mBio.02739-21.9 TABLE S1 Primers used in this study. Download Table S1, DOCX file, 0.03 MB. Copyright © 2022 Shi et al. 2022 Shi et al. https://creativecommons.org/licenses/by/4.0/ This content is distributed under the terms of the Creative Commons Attribution 4.0 International license.

Anti-Flag antibody (F1804), anti-MYC antibody (C3956), and anti-GSDMD antibody (G7422) were purchased from Sigma. Anti-HA antibody (3724) was purchased from Cell Signaling Technology. Anti-β-actin antibody was purchased from Abbkine (A01010). Anti-GSDMDC1 antibody (sc-393581) was purchased from Santa Cruz. The anti-PEDV N monoclonal antibody and the anti-GSDMD polyclonal antibody were prepared in our laboratory as previously described (26, 27). Necrosulfonamide (S8251) and disulfiram (S1680) were purchased from Selleck.

### Cytotoxicity assay.

Cell death was measured using a CytoTox 96 non-radioactive cytotoxicity assay kit (Promega) according to lactate dehydrogenase (LDH) released into medium.

### Immunoblotting.

Cells were harvested, lysed, subjected to SDS-PAGE using 8% to 12% SDS-PAGE gels (Fudebio, Hangzhou, China), and then transferred onto polyvinylidene difluoride membranes, followed by incubation with the indicated primary antibodies. The chemiluminescent signals were analyzed with a Clinx imaging system (Clinx Science Instruments).

### Propidium iodide assay.

HEK293T cells were seeded in 24-well plates and transfected with indicated plasmids for 24 h. The cells were stained with propidium iodide (BD Bioscience) and then analyzed with fluorescence microscopy.

### Flow cytometry assay.

Cells were harvested using trypsin, washed 3 times with PBS gently, and then stained with propidium iodide (BD Bioscience) according to the manufacturer’s instructions. The cells were analyzed with a flow cytometer (FACSVerse; Becton, Dickinson).

### RNA extraction and RT-qPCR.

Total RNA was extracted with RNA-easy isolation reagent (Vazyme Biotech Co., Ltd.). Reverse transcription was conducted with a HiScript III first-strand cDNA synthesis kit (+gDNA wiper) (Vazyme Biotech Co., Ltd.) according to the manufacturer’s instructions. Afterwards, cDNA samples were analyzed by qPCR using ChamQ universal SYBR qPCR master mix (Vazyme Biotech Co., Ltd.). Primers used for RT-qPCR are listed in Table S1C.

### Co-IP assay.

HEK293T cells seeded in 6-well plates were transfected with the specific plasmids for 24 h, and then cells were lysed with cell lysis buffer for immunoblotting and IP (Beyotime). The lysates were centrifuged at 4°C, and the supernatants were incubated with anti-Flag binding beads (M8823; Sigma) at 4°C overnight. The binding beads were then washed with TBS 5 times and then denatured in 1 × SDS-PAGE loading buffer for 10 min. Finally, the supernatants were analyzed by immunoblotting.

### Confocal immunofluorescence assay.

HEK293T cells were seeded in 24-well plates on coverslips, and after overnight culture indicated plasmids were transfected. At 24 h after transfection, cells were washed 3 times with cold PBS and then fixed with Immunol staining fix solution (Beyotime). The cells then were permeabilized with immunostaining permeabilization solution with saponin (Beyotime). After that, cells were blocked with QuickBlock blocking buffer for Immunol staining (Beyotime) and then incubated with primary antibody (C3956; anti-MYC; Sigma) at 4°C overnight. After washing 3 times with PBS, the cells were incubated with the secondary antibody (ab175471; goat anti-rabbit IgG Alexa Fluor 568; Abcam). Nuclei were stained with 4′,6-diamidino-2-phenylindole (DAPI) (Beyotime). The cells were then analyzed with a laser confocal microscope (IX81-FV1000; Olympus).

### Homology modeling.

Homology model of GSDMD was generated using SWISS-MODEL online software (https://swissmodel.expasy.org/). The full sequence of GSDMD used for homology modeling was obtained from NCBI (GenBank accession no. XM_021090506).

### Logo analysis.

The logo analysis of the predicted cleavage site of PEDV Nsp5 was generated by WebLogo (http://weblogo.threeplusone.com/). The templates used for analysis were polyproteins encoded by PEDV.

### Sequence alignment.

We collected amino acid sequences of pGSDMD and other GSDMD homologs from human (GenBank accession no. NP_001159709.1) and mouse (GenBank accession no. 6N9N_A) and those of Nsp5 of PEDV and Nsp5 of PDCoV (GenBank accession no. AKQ63081.1), SARS-CoV-2 (GenBank accession no. NC_045512), and MERS-CoV (GenBank accession no. NC_038294). SnapGene software was used to perform the multiple-sequence alignment.

### Statistical analysis.

All experiments were repeated three times or more. Data are presented as means ± standard deviations (SD) and analyzed by the two-tailed Student's *t* test or one-way analysis of variance (ANOVA) followed by Tukey’s multiple-comparison test by Prism software (GraphPad). The differences were considered significant when *P* values were <0.05 (*), <0.01 (**), and <0.001 (***).
