# Supplementary figures and images for: Coronaviruses Nsp5 Antagonizes Porcine Gasdermin D-Mediated Pyroptosis by Cleaving Pore-Forming p30 Fragment
Source: mBio. 2022 Jan 11;13(1):e02739-21. doi: 10.1128/mbio.02739-21 (PMC8749417; doi:10.1128/mbio.02739-21)

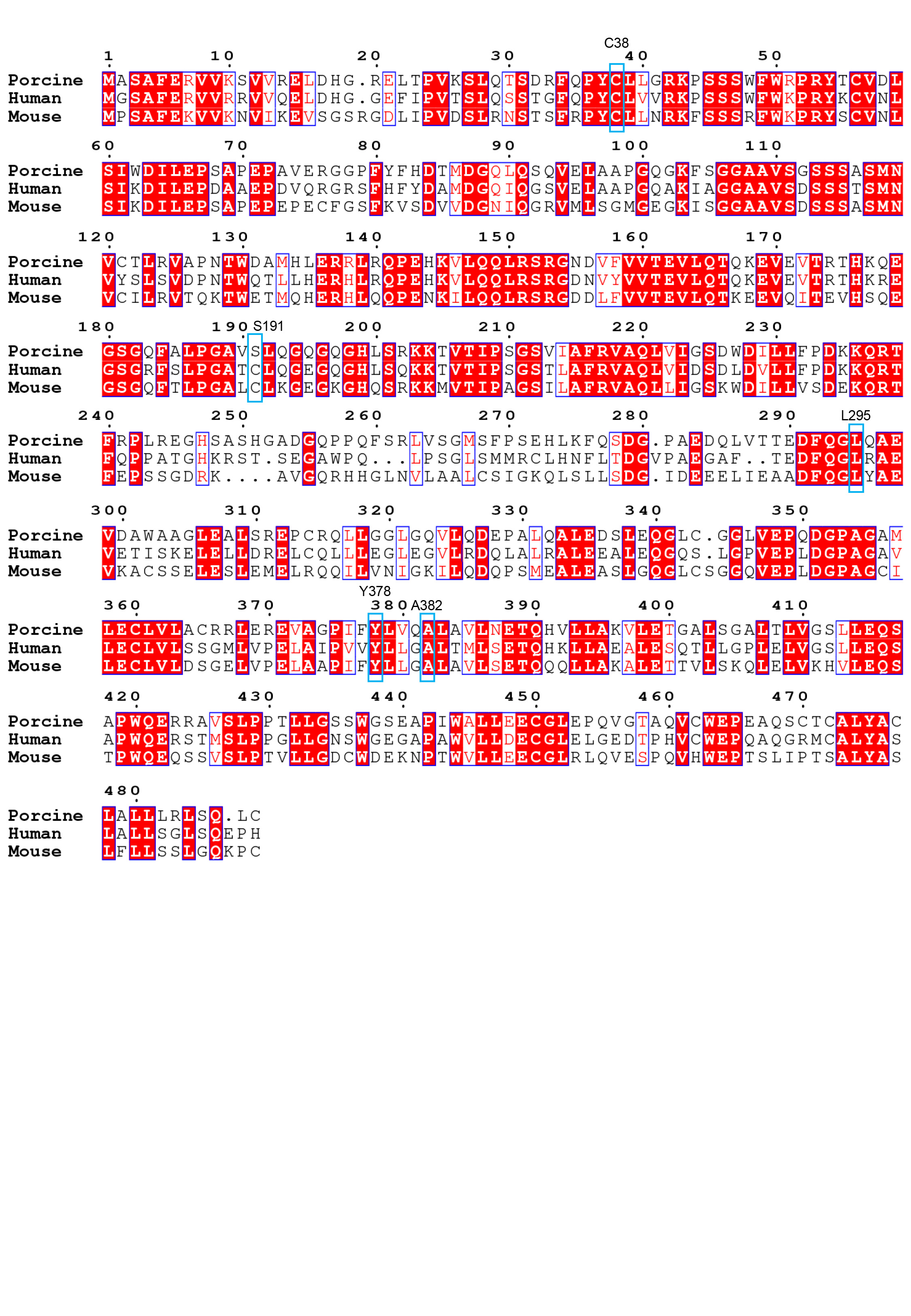

Supplement: FIG S1 [file mbio.02739-21-sf001.jpg]

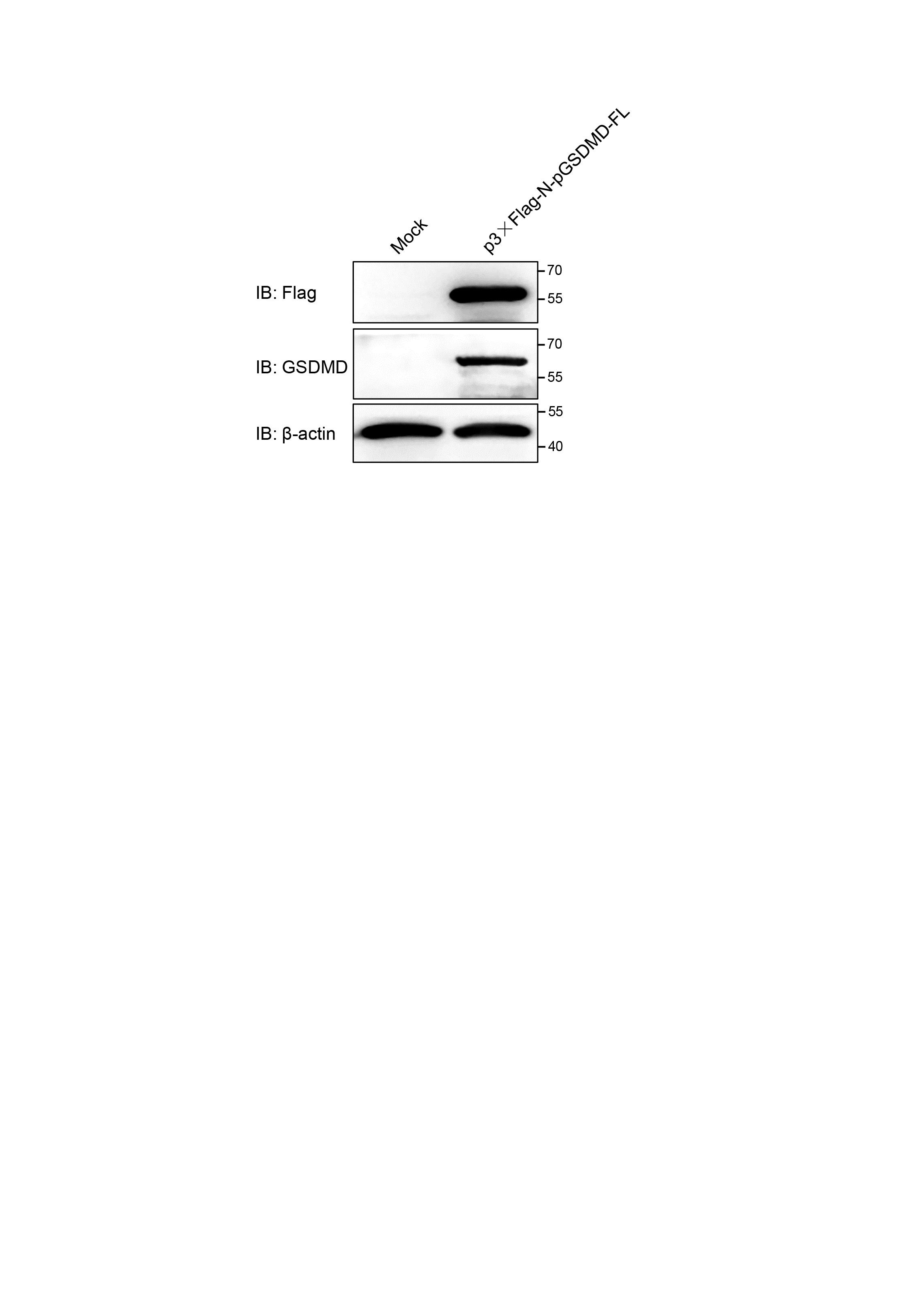

Supplement: FIG S2 [file mbio.02739-21-sf002.jpg]

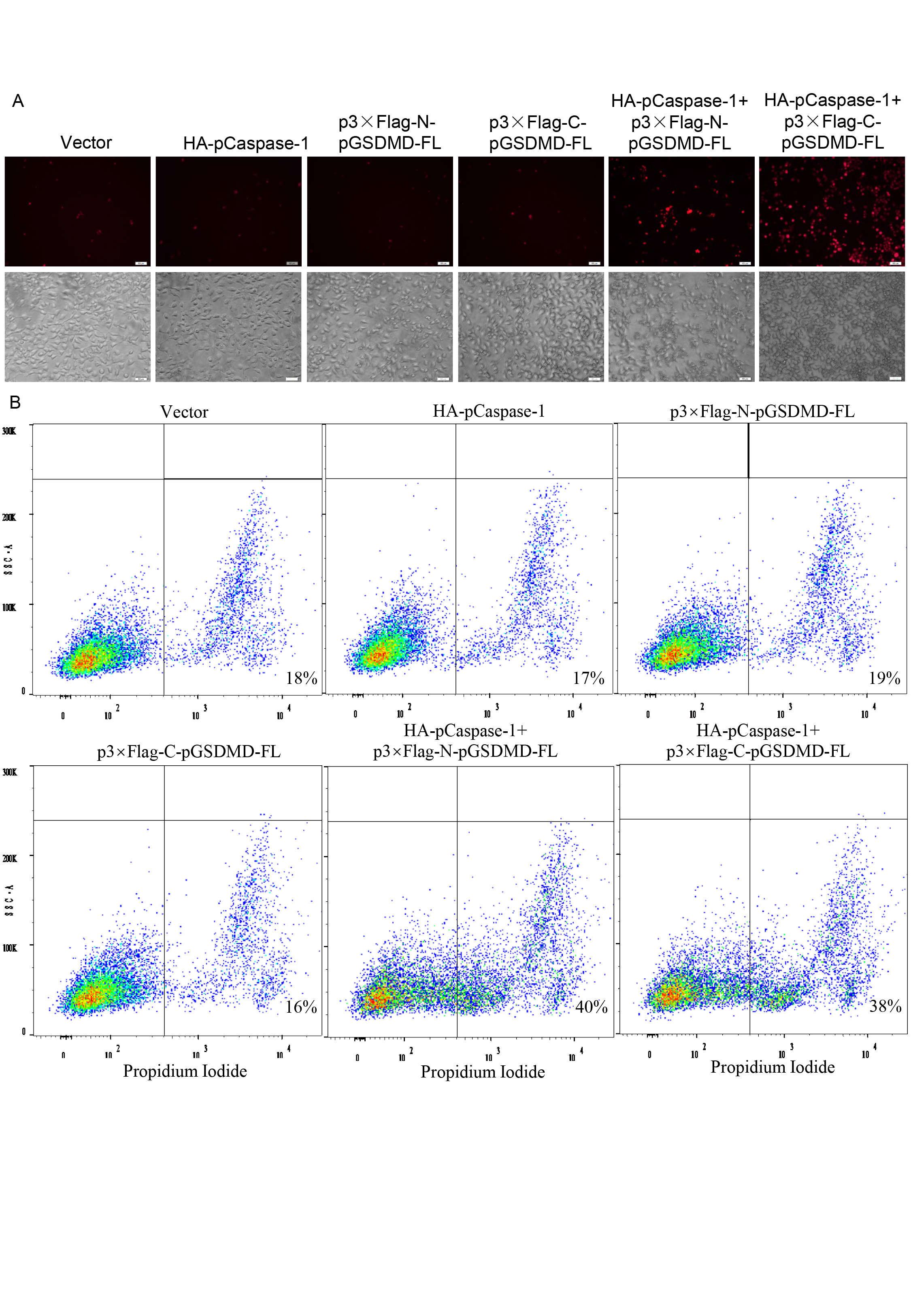

Supplement: FIG S3 [file mbio.02739-21-sf003.jpg]

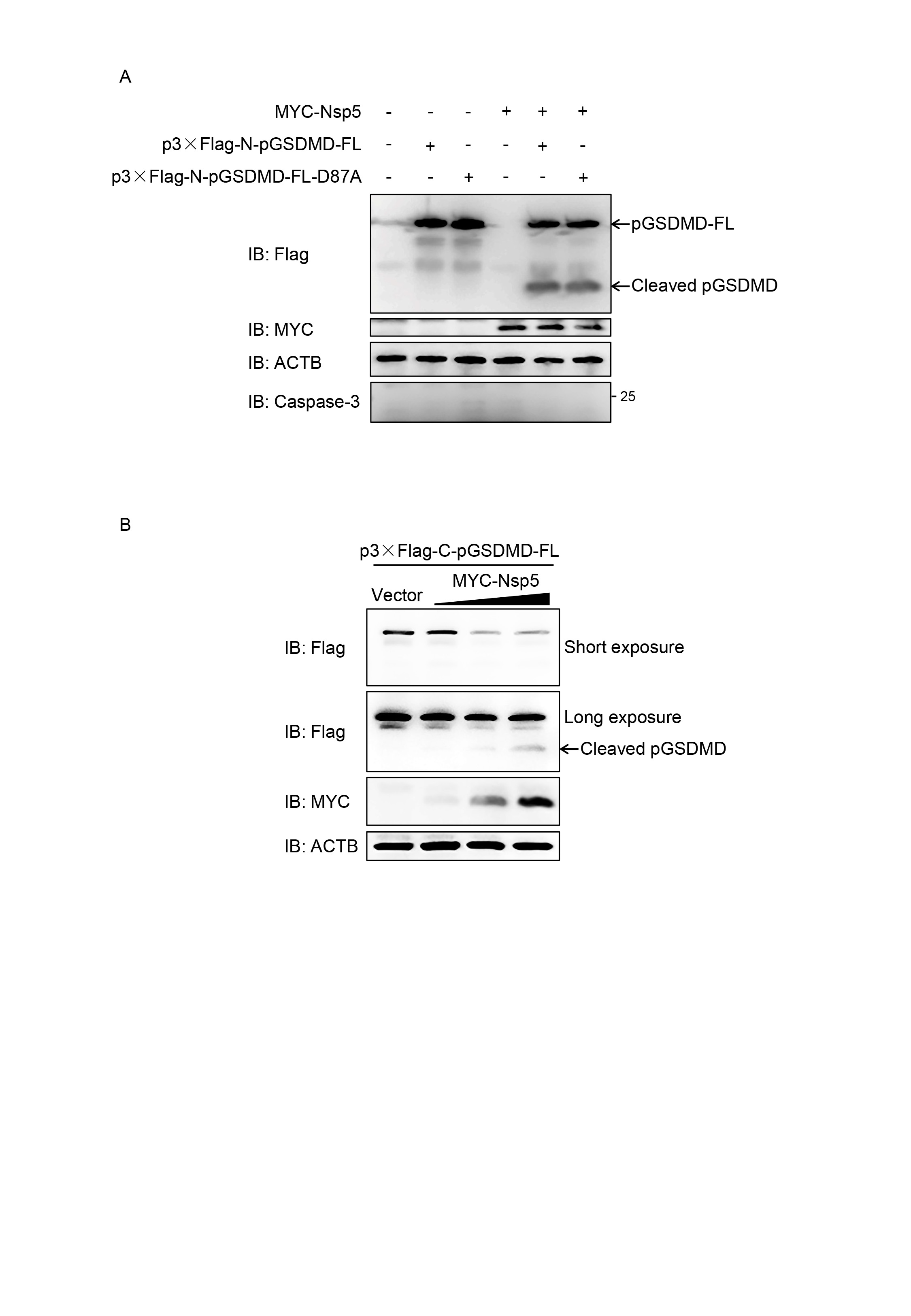

Supplement: FIG S4 [file mbio.02739-21-sf004.jpg]

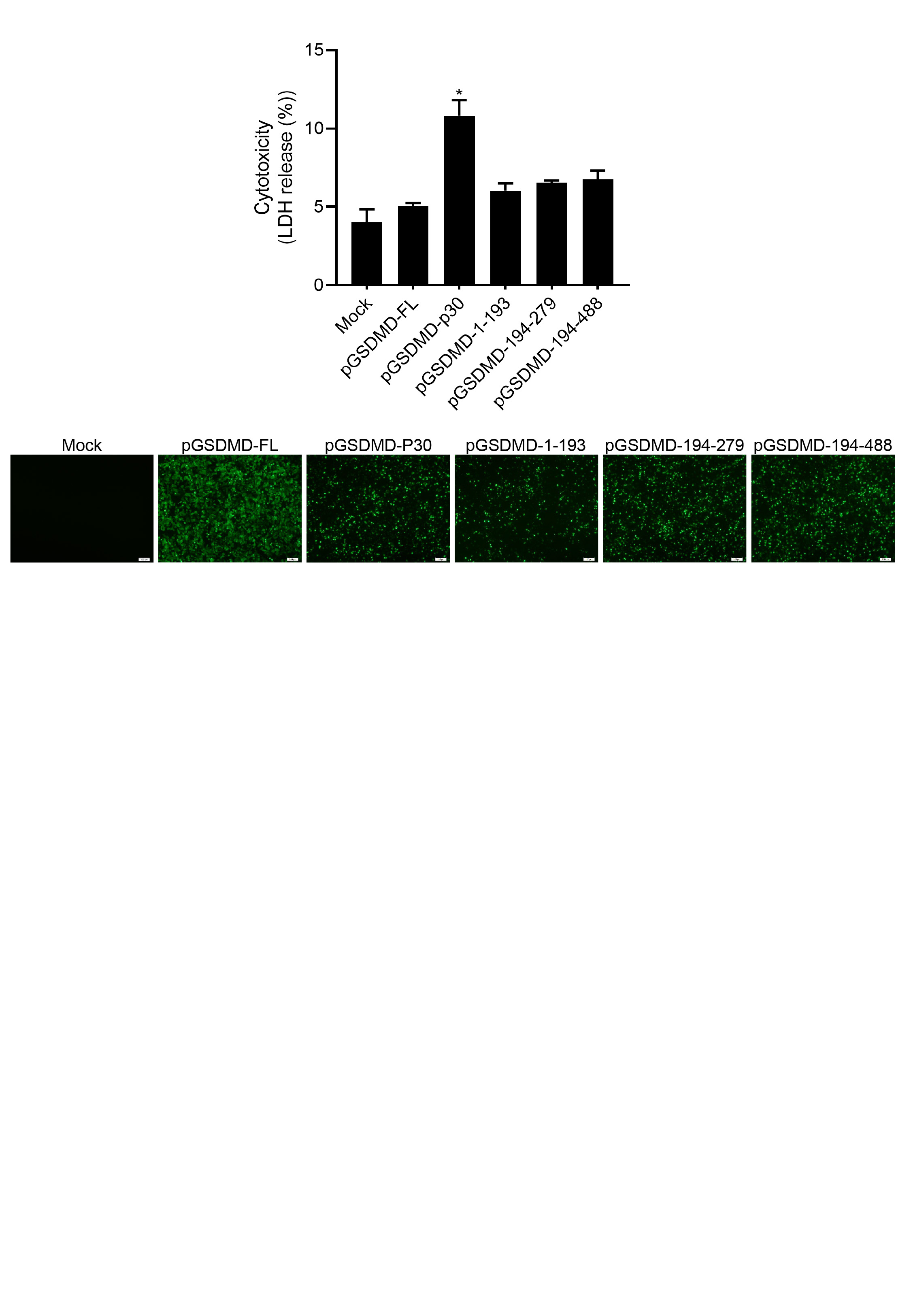

Supplement: FIG S5 [file mbio.02739-21-sf005.jpg]

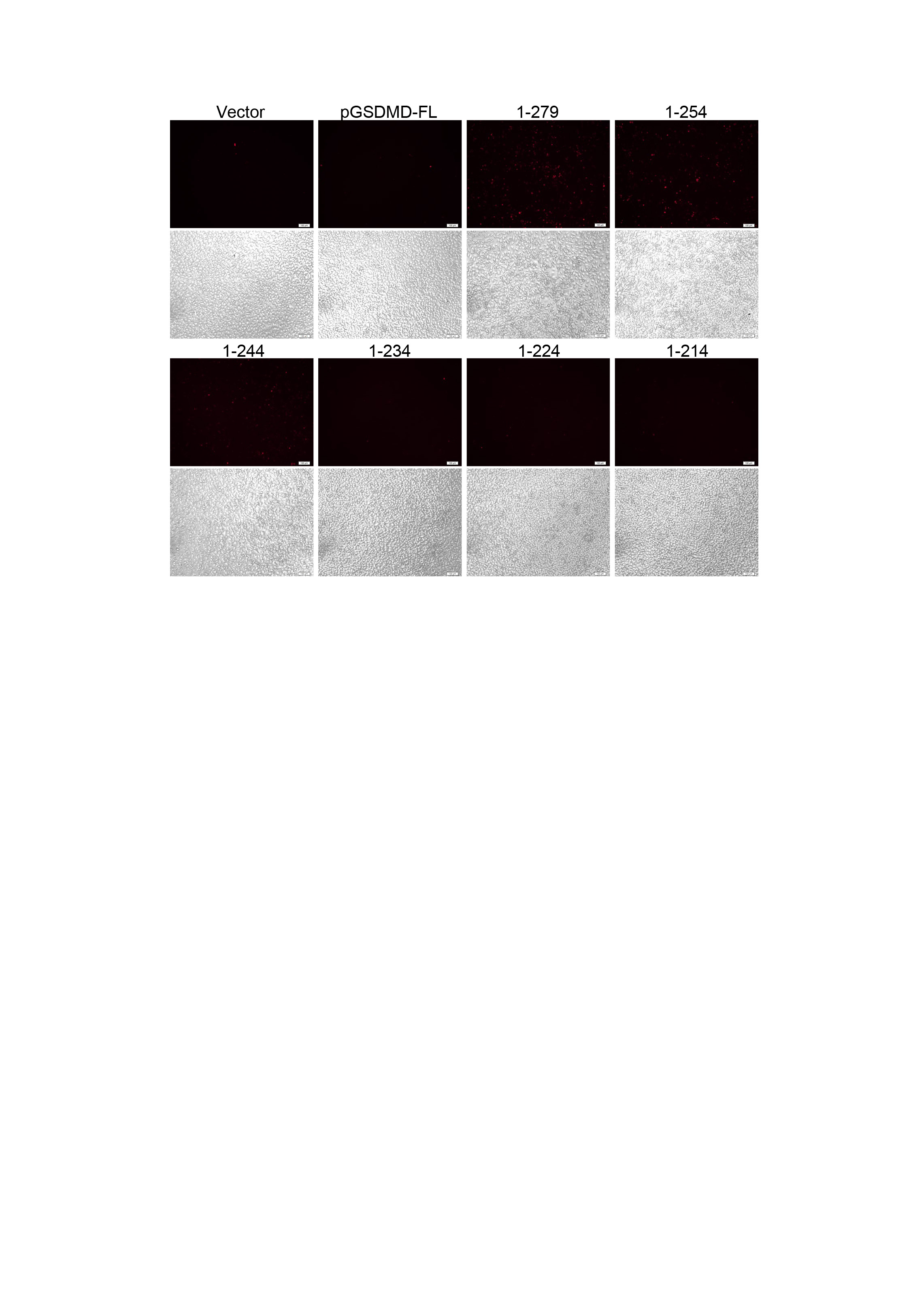

Supplement: FIG S6 [file mbio.02739-21-sf006.jpg]

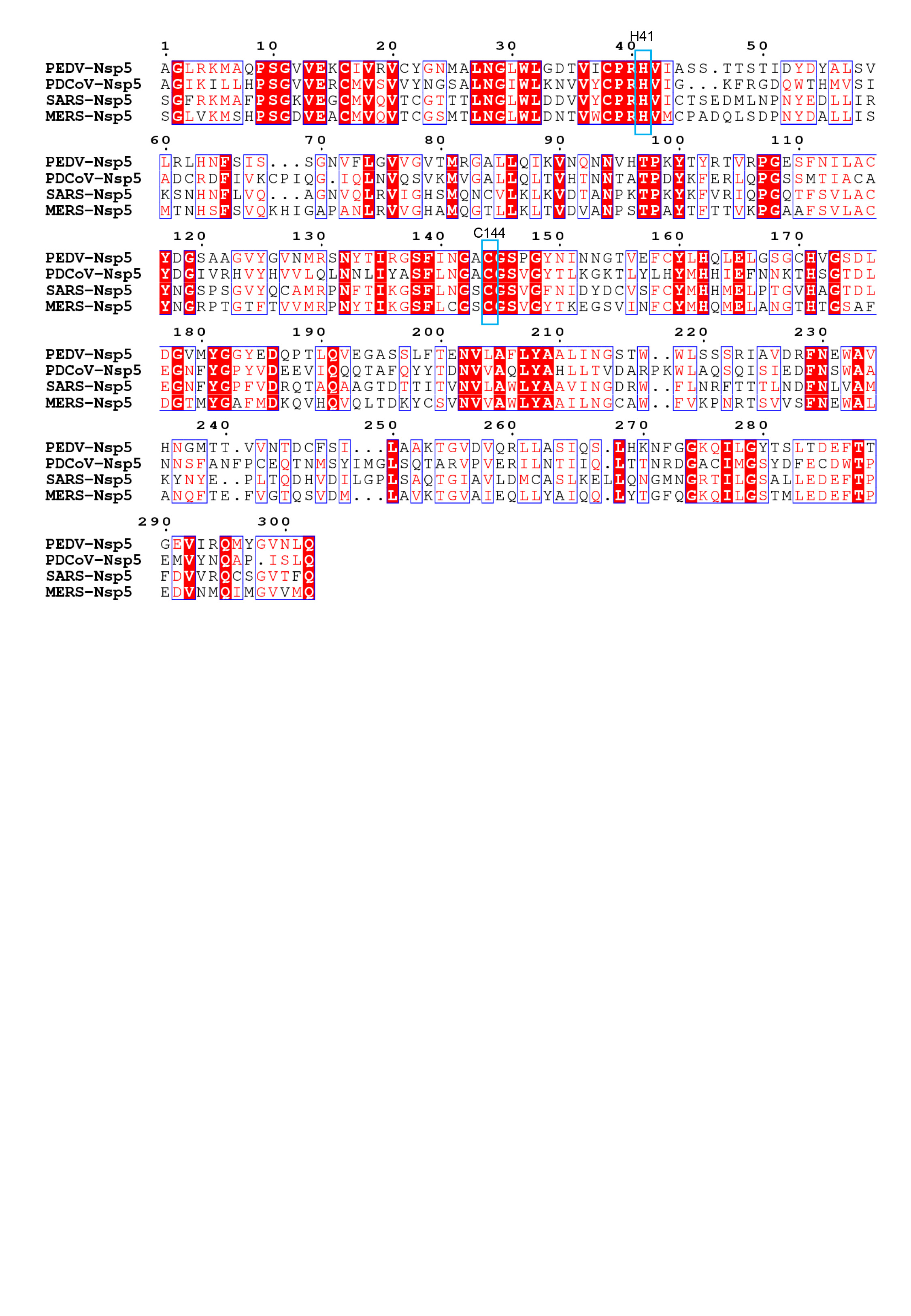

Supplement: FIG S7 [file mbio.02739-21-sf007.jpg]

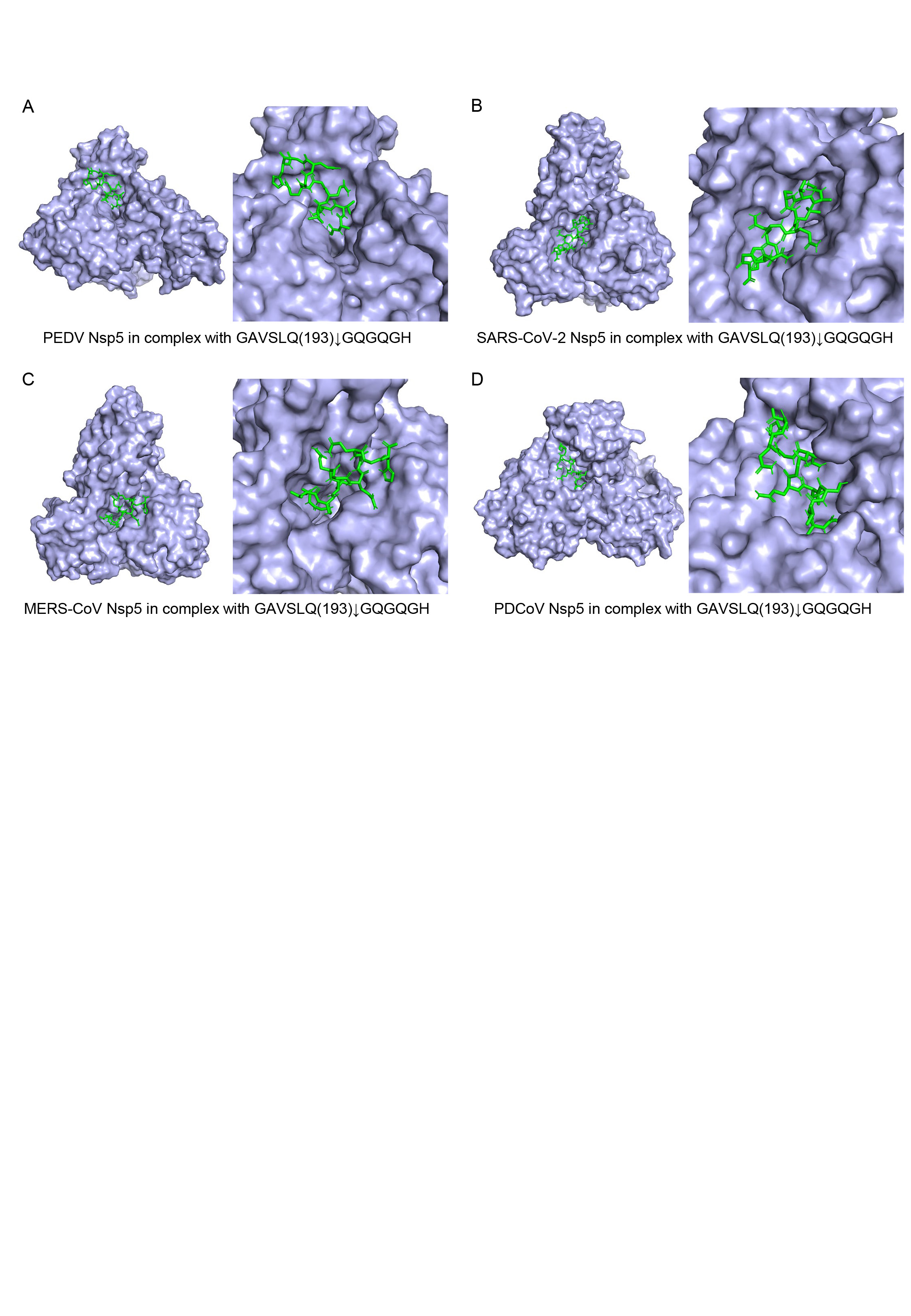

Supplement: FIG S8 [file mbio.02739-21-sf008.jpg]
