# Supplementary material for: Coronaviruses Nsp5 Antagonizes Porcine Gasdermin D-Mediated Pyroptosis by Cleaving Pore-Forming p30 Fragment
Source: mBio. 2022 Jan 11;13(1):e02739-21. doi: 10.1128/mbio.02739-21 (PMC8749417; doi:10.1128/mbio.02739-21)
Supplement: TABLE S1 [file mbio.02739-21-st001.docx]

**Supplementary table 1A** Primers used in this study for plasmids construction.

| **Primers** | **Sequences** |
| --- | --- |
| p3×Flag-N-GSDMD-FL forward | 5’-caagcttgcggccgcgaattctatggcatcagcctttgagagg-3’ |
| p3×Flag-N-GSDMD-FL reverse | 5’-tgccacccgggatcctctagactagcagagctggctgagcc-3’ |
| p3×Flag-C-GSDMD-FL forward | 5’-taagcttgcggccgcgaattcatggcatcagcctttgagagg-3’ |
| p3×Flag-C-GSDMD-FL reverse | 5’-gtcagcccgggatcctctagagcagagctggctgagcctc-3’ |
| HA-caspase-1 forward | 5’-ggaggcccgaattcggtcgaccatggccgataaggtgctga-3’ |
| HA-caspase-1 reverse | 5’-catgtctggatccccgcggccgcttaatgtcctgggaagagataaaaag-3’ |
| p3×Flag-C-GSDMD-p30 forward | 5’-taagcttgcggccgcgaattcatggcatcagcctttgagagg-3’ |
| p3×Flag-C-GSDMD-p30 reverse | 5’-gtcagcccgggatcctctagagtctgactggaacttcaggtgct-3’ |
| H-GSDMD-FL forward | 5’-aaggatgacgatgacaagcttatggggtcggcctttgag-3’ |
| H-GSDMD-FL reverse | 5’-atcagatctatcgatgaattcctagtggggctcctggctca-3’ |
| H-caspase-1 forward | 5’-caagcttgcggccgcgaattccatggccgacaaggtcctg-3’ |
| H-caspase-1 reverse | 5’-cctctagagtcgactggtaccttaatgtcctgggaagaggtagaaa-3’ |
| H-GSDMD-p30 forward | 5’-ccaagctggctagttaagcttatggggtcggcctttgag-3’ |
| H-GSDMD-p30 reverse | 5’-gagtttttgttcgaagggcccatctgtcaggaagttgtggaggc-3’ |
| MYC-PEDV-Nsp5 forward | 5’-ctgcacctcggttctatcgattgaattcgccaccatggctggcttgcgtaagatggc-3’ |
| MYC-PEDV-Nsp5 reverse | 5’-gccaagcttctgcaggtcgacttcacagatcctcttcagagatgagtttctgctcctgaagattaacgccat-3’ |
| p3×Flag-C-GSDMD-1-214aa forward | 5’-tgaaccgtcagaattaagcttatggcatcagcctttgagagg-3’ |
| p3×Flag-C-GSDMD-1-214aa reverse | 5’-tttgtagtcagcccgggatccaatgacgctgcccgaggg-3’ |
| p3×Flag-C-GSDMD-1-224aa forward | 5’-tgaaccgtcagaattaagcttatggcatcagcctttgagagg-3’ |
| p3×Flag-C-GSDMD-1-224aa reverse | 5’-tttgtagtcagcccgggatccaccaatcaccagctgggcc-3’ |
| p3×Flag-C-GSDMD-1-234aa forward | 5’-tgaaccgtcagaattaagcttatggcatcagcctttgagagg-3’ |
| p3×Flag-C-GSDMD-1-234aa reverse | 5’-tttgtagtcagcccgggatccgtccgggaagagaaggatgtcc-3’ |
| p3×Flag-C-GSDMD-1-244aa forward | 5’-tgaaccgtcagaattaagcttatggcatcagcctttgagagg-3’ |
| p3×Flag-C-GSDMD-1-244aa reverse | 5’-tttgtagtcagcccgggatccccgcagcggcctgaaggt-3’ |
| p3×Flag-C-GSDMD-1-254aa forward | 5’-taagcttgcggccgcgaattcatggcatcagcctttgagagg-3’ |
| p3×Flag-C-GSDMD-1-254aa reverse | 5’-gtcagcccgggatcctctagaatctgcaccgtgggaggc-3’ |
| p3×Flag-C-GSDMD-1-193 forward | 5’-tgaaccgtcagaattaagcttatggcatcagcctttgagagg-3’ |
| p3×Flag-C-GSDMD-1-193 reverse | 5’-tttgtagtcagcccgggatccctgcaaggacacagctccaggaagg-3’ |
| p3×Flag-C-GSDMD-194-279 forward | 5’-tgaaccgtcagaattaagcttatgggccagggccagggc-3’ |
| p3×Flag-C-GSDMD-194-279 reverse | 5’-tttgtagtcagcccgggatccgtctgactggaacttcaggtgct-3’ |
| p3×Flag-C-GSDMD-194-488 forward | 5’-tgaaccgtcagaattaagcttatgggccagggccagggc-3’ |
| p3×Flag-C-GSDMD-194-488 reverse | 5’-tttgtagtcagcccgggatccgcagagctggctgagcctc-3’ |
| p3×Flag-N-GSDMD-p30 forward | 5’-aaggatgacgatgacaagcttatggcatcagcctttgagagg-3’ |
| p3×Flag-N-GSDMD-p30 reverse | 5’-cagggatgccacccgggatcctcagtctgactggaacttcaggtg-3’ |
| p3×Flag-N-GSDMD-1-193 forward | 5’-aaggatgacgatgacaagcttatggcatcagcctttgagagg-3’ |
| p3×Flag-N-GSDMD-1-193 reverse | 5’-cagggatgccacccgggatcctcactgcaaggacacagctcc-3’ |
| p3×Flag-N-GSDMD-194-279 forward | 5’-aaggatgacgatgacaagcttggccagggccagggccac-3’ |
| p3×Flag-N-GSDMD-194-279 reverse | 5’-cagggatgccacccgggatcctcagtctgactggaacttcaggtg-3’ |
| p3×Flag-N-GSDMD-194-488 forward | 5’-aaggatgacgatgacaagcttggccagggccagggccac-3’ |
| p3×Flag-N-GSDMD-194-488 reverse | 5’-cagggatgccacccgggatccctagcagagctggctgagcc-3’ |
| pEGFP-C1-GSDMD-FL forward | 5’-tcagatctcgagctcaagcttttatggcatcagcctttgagagg-3’ |
| pEGFP-C1-GSDMD-FL reverse | 5’-ttatctagatccggtggatccctactgcaaggacacagctcca-3’ |
| pEGFP-C1-GSDMD-p30 forward | 5’-tcagatctcgagctcaagcttttatggcatcagcctttgagagg-3’ |
| pEGFP-C1-GSDMD-p30 reverse | 5’-ttatctagatccggtggatccttagtctgactggaacttcaggtgc-3’ |
| pEGFP-C1-GSDMD-1-193 forward | 5’-cagatctcgagctcaagcttttatggcatcagcctttgagagg-3’ |
| pEGFP-C1-GSDMD-1-193 reverse | 5’-ttatctagatccggtggatccctactgcaaggacacagctcca-3’ |
| pEGFP-C1-GSDMD-194-279 forward | 5’-tcagatctcgagctcaagcttttggccagggccagggcc-3’ |
| pEGFP-C1-GSDMD-194-279 reverse | 5’-ttatctagatccggtggatccctagtctgactggaacttcaggtgc-3’ |
| pEGFP-C1-GSDMD-194-488 forward | 5’-tcagatctcgagctcaagcttttggccagggccagggcc-3’ |
| pEGFP-C1-GSDMD-194-488 reverse | 5’-ttatctagatccggtggatccctagcagagctggctgagcc-3’ |
| MYC-C-SARS-CoV-2-Nsp5 forward | 5’-ctgcacctcggttctatcgattgaattcgccaccatgagtggttttagaaaaat-3’ |
| MYC-C-SARS-CoV-2-Nsp5 reverse | 5’-gccaagcttctgcaggtcgacttcacagatcctcttcagagatgagtttctgctcttggaaagtaacacctgagcat-3’ |
| MYC-C-MERS-CoV-Nsp5 forward | 5’-ctgcacctcggttctatcgattgaattcgccaccatgagcggtttggtgaaaatgt-3’ |
| MYC-C-MERS-CoV-Nsp5 reverse | 5’-gccaagcttctgcaggtcgacttcacagatcctcttcagagatgagtttctgctcctgcataaccacacccataat-3’ |
| MYC-C-PDCoV-Nsp5 forward | 5’-ctgcacctcggttctatcgattgaattcgccaccatggcaggtatcaaaatcctcct-3’ |
| MYC-C-PDCoV-Nsp5 reverse | 5’-gccaagcttctgcaggtcgacttcacagatcctcttcagagatgagtttctgctcctgcaatgaaattggagcct-3’ |
| MYC-N-SARS-CoV-2-Nsp5 forward | 5’-ctgcacctcggttctatcgattgaattcgccaccatggagcagaaactcatctctgaagaggatctgagtggttttagaaaaatggcat-3’ |
| MYC-N-SARS-CoV-2-Nsp5 reverse | 5’-gccaagcttctgcaggtcgacttcattggaaagtaacacctgagc-3’ |
| MYC-N-PDCoV-Nsp5 forward | 5’-ctgcacctcggttctatcgattgaattcgccaccatggagcagaaactcatctctgaagaggatctggcaggtatcaaaatcctcct-3’ |
| MYC-N-PDCoV-Nsp5 reverse | 5’-gccaagcttctgcaggtcgacttcactgcaatgaaattggagc-3’ |

**Supplementary table 1B** Primers used in this study for point mutations.

| **Primers** | **Sequences** |
| --- | --- |
| p3×Flag-C-GSDMD-D254A forward | 5’-cctcccacggtgcagccgggcagccgccgcag-3’ |
| p3×Flag-C-GSDMD-D254A reverse | 5’-tgcggcggctgcccggctgcaccgtgggag-3’ |
| p3×Flag-C-GSDMD-D279A forward | 5’-gcacctgaagttccagtcagccgggcccgcggaggaccagc-3’ |
| p3×Flag-C-GSDMD-D279A reverse | 5’-gctggtcctccgcgggcccggctgactggaacttcaggtgc-3’ |
| p3×Flag-C-GSDMD-p30-C38A forward | 5’-agccctacgcccttctgggcaggaagccctcg-3’ |
| p3×Flag-C-GSDMD-p30-C38A reverse | 5’-cagaagggcgtagggctggaagcggtcagagg-3’ |
| p3×Flag-C-GSDMD-p30-S191A forward | 5’-agctgtggccttgcagggccagggccagggcc-3’ |
| p3×Flag-C-GSDMD-p30-S191A reverse | 5’-cctgcaaggccacagctccaggaagggcaaac-3’ |
| p3×Flag-C-GSDMD-FL-L295D forward | 5’-aagacttccagggcgaccaggccgaggtggacgcc-3’ |
| p3×Flag-C-GSDMD-FL-L295D reverse | 5’-gtcgccctggaagtcttcggtggtcaccagct-3’ |
| p3×Flag-C-GSDMD-FL-Y378D forward | 5’-catcttcgacctggtacaggctctagctgtgct-3’ |
| p3×Flag-C-GSDMD-FL-Y378D reverse | 5’-gtaccaggtcgaagatggggccggcgacttcc-3’ |
| p3×Flag-C-GSDMD-FL-A382D forward | 5’-ggtacaggatctagctgtgctgaatgaaaccca-3’ |
| p3×Flag-C-GSDMD-FL-A382D reverse | 5’-cagctagatcctgtaccaggtagaagatggggc-3’ |
| p3×Flag-C-GSDMD-FL-L295D+Y378D forward | 5’-ccatcttcgacctggtacaggctctagctgtg-3’ |
| p3×Flag-C-GSDMD-FL-L295D+Y378D reverse | 5’-taccaggtcgaagatggggccggcgacttccc-3’ |
| p3×Flag-C-GSDMD-FL-L295D+Y378D+A382D forward | 5’-gacctagctgtgctgaatgaaacccagcatgtg-3’ |
| p3×Flag-C-GSDMD-FL-L295D+Y378D+A382D reverse | 5’-attcagcacagctaggtcctgtaccaggtcgaagatggg-3’ |
| MYC-PEDV-Nsp5-H41A forward | 5’-gcgctgttatagcgtctagtactactagcactataga-3’ |
| MYC-PEDV-Nsp5-H41A reverse | 5’-tagacgctataacagcgcgtgggcagataacagtatca-3’ |
| MYC-PEDV-Nsp5-C144A forward | 5’-ttaatggcgctgctggttcacctggttataacattaaca-3’ |
| MYC-PEDV-Nsp5-C144A reverse | 5’-aaccagcagcgccattaatgaacgagcctctaa-3’ |
| p3×Flag-N-GSDMD-FL-Q193A forward | 5’-tggagctgtgtccttggcgggccagggccagggcca-3’ |
| p3×Flag-N-GSDMD-FL-Q193A reverse | 5’-gccaaggacacagctccaggaagggcaaactgg-3’ |
| p3×Flag-N-GSDMD-FL-Q195A forward | 5’-tgtgtccttgcagggcgcgggccagggccacctgag-3’ |
| p3×Flag-N-GSDMD-FL-Q195A reverse | 5’-gcgccctgcaaggacacagctccaggaagggca-3’ |
| p3×Flag-N-GSDMD-FL-Q197A forward | 5’-agggcgcgggccacctgagccggaagaagacgg-3’ |
| p3×Flag-N-GSDMD-FL-Q197A reverse | 5’-tcaggtggcccgcgccctggccctgcaaggaca-3’ |
| p3×Flag-C-GSDMD-p30-K235D forward | 5’-gggacatccttctcttcccggacgacaagcagcgaaccttcaggccgct-3’ |
| p3×Flag-C-GSDMD-p30-K235D reverse | 5’-agcggcctgaaggttcgctgcttgtcgtccgggaagagaaggatgtccc-3’ |
| p3×Flag-C-GSDMD-p30-K236D forward | 5’-acatccttctcttcccggacaaggaccagcgaaccttcaggccgctgcg-3’ |
| p3×Flag-C-GSDMD-p30-K236D reverse | 5’-cgcagcggcctgaaggttcgctggtccttgtccgggaagagaaggatgt-3’ |
| p3×Flag-C-GSDMD-p30-Q237D forward | 5’-ccttctcttcccggacaagaaggaccgaaccttcaggccgctgcggg-3’ |
| p3×Flag-C-GSDMD-p30-Q237D reverse | 5’-cccgcagcggcctgaaggttcggtccttcttgtccgggaagagaagg-3’ |
| p3×Flag-C-GSDMD-p30-R238D forward | 5’-cttcccggacaagaagcaggacaccttcaggccgctgcggg-3’ |
| p3×Flag-C-GSDMD-p30-R238D reverse | 5’-cccgcagcggcctgaaggtgtcctgcttcttgtccgggaag-3’ |
| p3×Flag-C-GSDMD-p30-T239D forward | 5’-cttcccggacaagaagcagcgagacttcaggccgctgcgggaaggc-3’ |
| p3×Flag-C-GSDMD-p30-T239D reverse | 5’-gccttcccgcagcggcctgaagtctcgctgcttcttgtccgggaag-3’ |
| p3×Flag-C-GSDMD-p30-F240D forward | 5’-cggacaagaagcagcgaaccgacaggccgctgcgggaaggcc-3’ |
| p3×Flag-C-GSDMD-p30-F240D reverse | 5’-ggccttcccgcagcggcctgtcggttcgctgcttcttgtccg-3’ |
| p3×Flag-C-GSDMD-p30-R241D forward | 5’-ggacaagaagcagcgaaccttcgacccgctgcgggaaggccatagcg-3’ |
| p3×Flag-C-GSDMD-p30-R241D reverse | 5’-cgctatggccttcccgcagcgggtcgaaggttcgctgcttcttgtcc-3’ |
| p3×Flag-C-GSDMD-p30-P242D forward | 5’-agaagcagcgaaccttcagggacctgcgggaaggccatagcgc-3’ |
| p3×Flag-C-GSDMD-p30-P242D reverse | 5’-gcgctatggccttcccgcaggtccctgaaggttcgctgcttct-3’ |
| p3×Flag-C-GSDMD-p30-L243D forward | 5’-gcagcgaaccttcaggccggaccgggaaggccatagcgcct-3’ |
| p3×Flag-C-GSDMD-p30-L243D reverse | 5’-aggcgctatggccttcccggtccggcctgaaggttcgctgc-3’ |
| p3×Flag-C-GSDMD-p30-R244D forward | 5’-cagcgaaccttcaggccgctggacgaaggccatagcgcctcccac-3’ |
| p3×Flag-C-GSDMD-p30-R244D reverse | 5’-gtgggaggcgctatggccttcgtccagcggcctgaaggttcgctg-3’ |
| MYC-SARS-CoV-2-Nsp5-H41A forward | 5’-gtccaagagctgtgatctgcacctctgaagaca-3’ |
| MYC-SARS-CoV-2-Nsp5-H41A reverse | 5’-agatcacagctcttggacagtaaactacgtcatcaa-3’ |
| MYC-SARS-CoV-2-Nsp5-C145A forward | 5’-atggttcagctggtagtgttggttttaacatagattatg-3’ |
| MYC-SARS-CoV-2-Nsp5-C145A reverse | 5’-cactaccagctgaaccattaaggaatgaaccctt-3’ |
| MYC-MERS-CoV-Nsp5-H41A forward | 5’-gcccacgagccgtaatgtgcccggctgaccagt-3’ |
| MYC-MERS-CoV-Nsp5-H41A reverse | 5’-acattacggctcgtgggcaccagactgtgttgt-3’ |
| MYC-MERS-CoV-Nsp5-C148A forward | 5’-tgtggttctgctggtagtgttggttacaccaagga-3’ |
| MYC-MERS-CoV-Nsp5-C148A reverse | 5’-actaccagcagaaccacacagaaaggaaccctt-3’ |
| MYC-PDCoV-Nsp5-H41A forward | 5’-cacgcgctgtaattggaaaattccgtggtgacc-3’ |
| MYC-PDCoV-Nsp5-H41A reverse | 5’-ttccaattacagcgcgtgggcagtagacaacatt-3’ |
| MYC-PDCoV-Nsp5-C144A forward | 5’-ttaacggagctgctggtagtgtgggttacactcttaagg-3’ |
| MYC-PDCoV-Nsp5-C144A reverse | 5’-taccagcagctccgttaaggaagcttgcataaa-3’ |

**Supplementary table 1C** Primers used in this study for qPCR.

| **Primers** | **Sequences** |
| --- | --- |
| PEDV-N forward | 5’-cggaacaggacctcacgcc-3’ |
| PEDV-N reverse | 5’-acaatctcaactacgctgggaag-3’ |
| PEDV S1 forward | 5’-cggtttgttggatgctgtc-3’ |
| PEDV S1 reverse | 5’-aataaagaatacgctgaatggc-3’ |
| GAPDH forward | 5’-cactgaggaccaggttgtgtcctgtgac-3’ |
| GAPDH reverse | 5’-tccaccaccctgttgctgtagccaaattc-3’ |
